# Parental and social factors in relation to child psychopathology, behavior, and cognitive function

**DOI:** 10.1038/s41398-020-0761-6

**Published:** 2020-02-26

**Authors:** Han Zhang, Zu Xuan Lee, Tonya White, Anqi Qiu

**Affiliations:** 1 Department of Biomedical Engineering, National University of Singapore, Singapore, Singapore; 2 Department of Child and Adolescent Psychiatry, Erasmus University Medical Center - Sophia, Children’s Hospital, Rotterdam, The Netherlands

**Keywords:** Human behaviour, Predictive markers

## Abstract

Parental and social factors have long-term impact on the neurodevelopment of offspring, but tend to highly covary with each other. Thus, it is difficult to parse out which parental and social factor contributes most to neurodevelopmental outcomes. This study aimed to assess clusters of parental and social factors associated with child psychopathology, behavioral problems, and cognition. This study employed the data of 11,875 children (9 to 11 years) from the Adolescent Brain Cognitive Development (ABCD) study. Principal component analysis (PCA) was performed on 39 environmental measures and 30 child behavior and cognitive measures separately to identify clusters of parental and social factors and clusters of child psychopathology, behaviour, and cognition. Regression analysis was used to examine independent effects of each cluster of parental and social factors on child psychopathology, behavioral problems, and cognition. Greater *Parent Psychopathology* cluster was associated with greater *Child Psychopathology* cluster. Moreover, greater *Socioeconomic Status* cluster was associated with greater child *General Cognition* and *Executive Function* but less *Behavioral Inhibition* clusters. Greater *Proximal Social Environment and Interaction* cluster were associated with less child *Impulsive Behavior* and *Behavioral Inhibition*, but greater *Behavioral Activation* cluster. The environmental clusters related to birth outcomes, maternal tobacco, and drug use were not significantly related to child psychopathology, behavior, and cognition. Our findings suggest that socioeconomic status, parental psychopathology, and social environment and interactions are the strongest risks for behavioral problems and cognitive performance in a general child population. Intervention programs should target modifiable factors within these domains.

## Introduction

Parental, socioeconomic, and social factors, such as parent psychopathology, pregnancy complications, household income, parental education, and family environment, can have long-term impact on the neurodevelopment of offspring^1–4^. However, most of existing studies typically assess parental, socioeconomic, and social factors, and their influences on child psychopathology, behavior, and cognition, separately. These environmental factors not only play an important role in neurodevelopment, but also tend to covary highly with each other, which makes it difficult to parse out which parental and social factor contributes most to neurodevelopmental outcomes, or whether the risk is additive^5,6^.

The Adolescent Brain Cognitive Development (ABCD) study (version 2.0) acquired comprehensive information on prenatal and postnatal parental, socioeconomic, and social environment as well as child outcomes in 11,875 children aged at 9 to 11 years^7^. It provided a unique opportunity to assess each aspect of parental, socioeconomic, and psychosocial factors in relation with child psychopathology, behavioral problems, and cognition when considering the interplay of different aspects of parental and social factors. For this, we employed principal component analysis to identify clusters within a wide spectrum of parental, socioeconomic, and social environmental factors and clusters within a wide spectrum of child psychopathology, behavioral problems, and cognition. Such an approach provides a comprehensive map for understanding the contribution of individual aspects of parental, socioeconomic and social factors to child psychopathology, behavioral problems, and cognition, which potentially provides the guidance of future intervention on improving child neurodevelopment in a general population.

## Methods

### Participants

Participant data were obtained from the open baseline from the ongoing Adolescent Brain Cognitive Development (ABCD) study (release 2.0; https://abcdstudy.org/). Youth (*n* = 11,875) 9–11 years of age were recruited for this study and formed a similar proportion of males and females living in the United States. The sample selection criteria were targeted to reflect the sociodemographic proportion of the U.S. population as described in the ABCD study design^7^. All participants were administered assessments to obtain data on the respective youth’s brain morphology, cognitive function, substance use, demographics, and environment^8^. Written informed consent was obtained from all parents, and all children provided assent to a research protocol approved by the institutional review board at each data collection site (https://abcdstudy.org/study-sites/)^9^.

Of the 11,875 participants, we excluded 23 subjects with missing values of demographics, 3219 subjects with one or more missing values of the parental and social environmental measures, 914 subjects with one or more missing values of the questionnaires/tasks of the child psychopathology, behavior, and cognitive measures, and 283 subjects with missing values in either of these two data. Therefore, our study employed 11,875 participants and 8002 participants (67.4% of full sample) for statistical analysis, separately. Supplementary Table S1 in the Supplementary Material lists the subject id whose data were not included in this study.

### Parental and social environmental measures

This study included 39 parental, socioeconomic, and social environmental measures, including10 measures of parent psychopathology, 6 maternal substance use measures, 5 developmental adversity measures, 7 social demographics, 5 proximal environmental measures, and 6 social interaction measures^8,10^.

### Parent psychopathology

Parent psychopathology symptoms were assessed using the Adult Self Report (ASR) and Family History Assessment Module Screener (FHAM-S) questionnaires. The ASRprovides 8 empirically-based syndrome scales (anxious/depressed, withdrawn, somatic complaints, thought problems, attention problems, aggressive behaviour, rule-breaking behavior, and intrusive)^11^. FHAM-S reports the presence/absence of symptoms associated with alcohol and drug use, depression, and mania in all 1st and 2nd degree “blood relatives” of the youth^12^. The presence of alcohol and drug use problems of the child’s relatives was defined as the family psychopathology risk of substance use disorders. Similarly, the accumulated presence of depression and mania was scored as the family psychopathology risk of mental disorders.

### Maternal substance use

The parent-reported Developmental History Questionnaire was used to assess maternal consumption of tobacco, alcohol, and marijuana before and after the mothers knew that they were pregnancy^13–15^.

### Developmental adversity

The developmental History Questionnaire^8^ was used to assess prematurity, birth weight, pregnancy and birth complications and the Modified Ohio State University Traumatic Brain Injury Screen-Short Version^16^ was employed to assess the parent-report overall brain injury/concussion during the child’s development.

### Social demographics

The parent-report demographics battery from the PhenX toolkit measured social demographics of the parental highest education, household annual income, and marriage status^17^. Economic insecurity^18^, the grand total Uniform Crime Reports, Area Deprivation Index by the scaled weighted sum, and the estimated lead risk in census tract of primary residential address^19,20^ were also employed to provide additional information about socioeconomic influences.

### Proximal environment

The “Safety from Crime” items from the PhenX Toolkit was used to assess neighborhood safety and crime reports^21,22^. Additionally, children reported their school risk and protective factors via a 12-item Inventory for School Risk and Protective Factors of the PhenX toolkit^23^. Three measures was selected to assess a child’s connectedness to his/her school, including school teacher and classroom environment, personal involvement in school, and alienation from academic goals.

### Social interaction

The child-reported parental monitoring and acceptance, as well as the child- and parent-reported prosocial tendency and family conflicts were included to measure social interactions. Parent monitoring was accessed by a 5-item summary score of the Parental Monitoring Scale^24^. Parent acceptance was evaluated by the Acceptance Scale, a subscale of the Child Report of Behavior Inventory (CRPBI)^25^. Prosocial behavior (e.g., being nice, helping, caring) was assessed using the Prosocial Behavior Scale, a subscale from the “Strengths and Difficulties Questionnaire” (SDQ)^26^. Both parents and youth reported on the youth’s prosocial behavior (e.g., being considerate of other people’s feelings, often offering to help others). In order to assess the family conflicts, the ABCD protocol utilized a 9-item Family Conflict subscale of the Moos Family Environment Scale (FES) for the baseline protocol^27^.

**Fig. 1:**
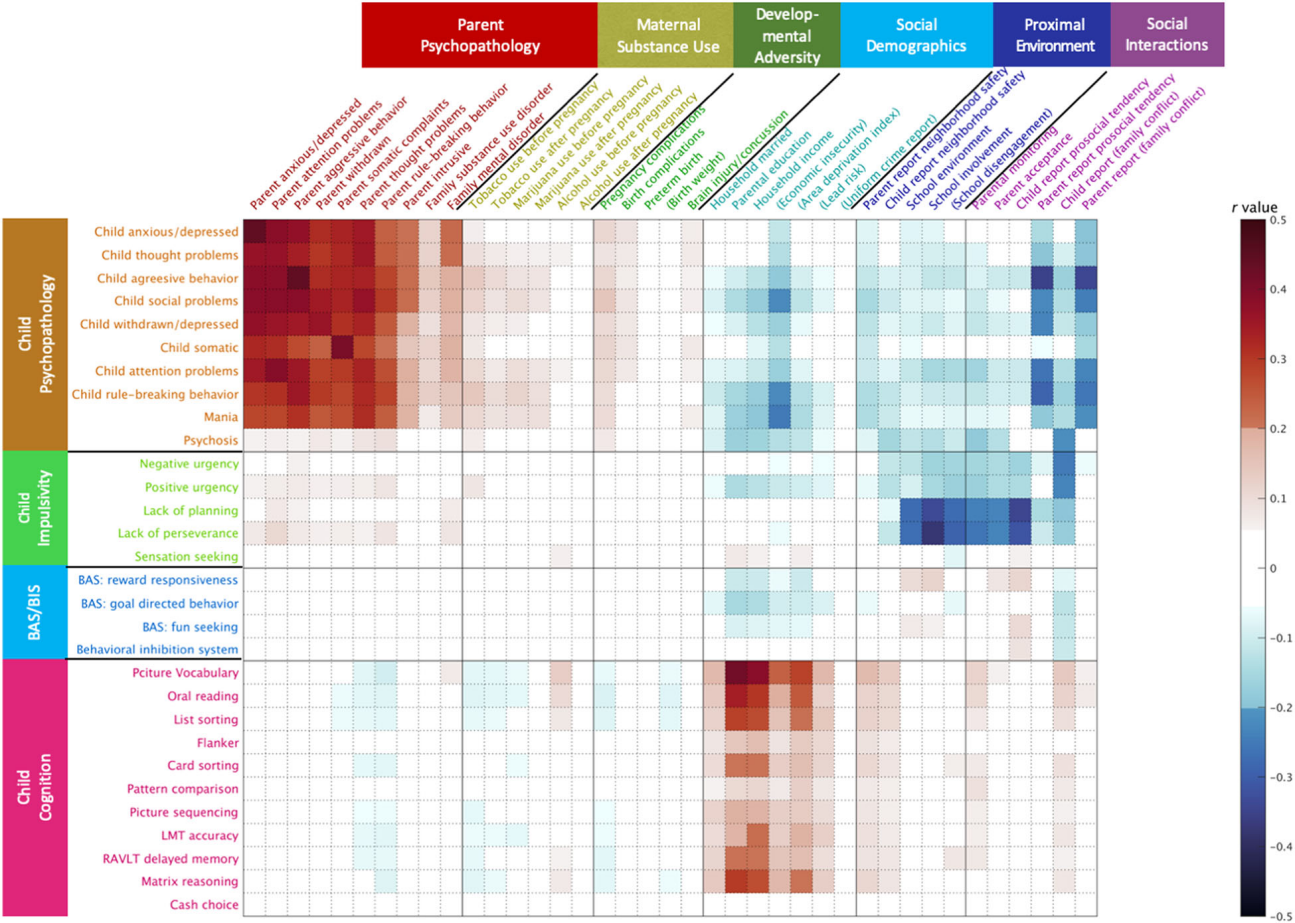
Correlation heat map. The value in the color bar corresponds to Pearson correlation coefficient. Significant correlations are shown in non-white color at Bonferroni corrected *p* < 0.001. The scores of the variables in (•) were inversed in order to align their direction with the variables in each domain.

For the parental and environmental measures related to psychopathology, maternal substance use, and developmental adversity, higher scores represents more severe psychiatric symptoms, worse substance use, and developmental adversity. For the measures of social demographics, proximal environment, and social interactions, higher scores represent better socioeconomic status, proximal environment, and social interactions. For the ease of interpretation, a few scores were inverted to align the direction in their same category as mentioned above. Figure 1 marks these inverted measures in parenthesis.

### Child psychopathology, behavior, and cognition

This study employed 30 child psychopathology, behavior, and cognitive measures, including 10 child psychopathology measures, 9 behavior measures, and 11 cognitive measures^8,28^. To provide converging evidence about the youth’s behavior, we also utilized the available data (*n* = 2440) with the teach-reported total behavior problems which were evaluated by the Brief Problem Monitor-Teacher Form^11^.

### Child psychopathology

Child Psychopathology was assessed based on the parent report of Child Behavior Checklist (CBCL)^11^, the ten-item Mania Scale derived from the Parent General Behavior Inventory for Children and Adolescents^29^, and the Prodromal Questionnaire Brief Version^30^. This study included 8 empirically-based syndrome scales from CBCL (aggressive behavior, anxious/depressed, attention problems, rule-breaking behavior, somatic complaints, social problems, thought problems, and withdrawn/depressed scales), a risk score of bipolar variability in mood and behaviour, and a severity score of psychosis risk symptoms.

### Child behavior

The 20-item Children-Short Form (UPPS-P) was used to assess five facets of impulsivity^31^, including negative and positive urgency, lack of planning, lack of perseverance, and sensation seeking. The 24-item Behavioral Inhibition/Activation Scales (BIS/BAS) were also utilized: BIS (e.g., worry, fearfulness), BAS drive (intensity of goal directed behavior), BAS reward responsiveness (excitement over reinforcing outcomes), and BAS fun seeking (enjoyment for its own sake, spontaneity)^32^.

### Child cognition

The neurocognitive battery comprised of 11 tasks^28^ and was administered using an iPad with one-on-one monitoring by a research assistant. Among the 11 cognitive tasks, there were 7 from the NIH Toolbox (http://www.nihtoolbox. org), including flanker (inhibitory control), dimensional change card sort (cognitive flexibility), list sorting working memory (working memory), picture sequence memory (episodic memory), pattern comparison processing speed (processing speed), picture vocabulary (vocabulary comprehension), and oral reading recognition tasks (reading decoding). ABCD also administered Matrix Reasoning Task from the Wechsler Intelligence Test for Children-V (fluid Reasoning)^33^, Little Man Task (LMT, visual-spatial processing), Rey Auditory Verbal Learning Test (RAVLT, auditory learning, memory, and recognition), and Cash Choice Task (a single-item delayed gratification measure with dichotomous scoring). Notably, we employed the response accuracy of LMT, the delayed recall accuracy of RAVLT, and the total scaled score of Matrix Reasoning.

For measures related to child psychopathology and behavior, a higher score represented worse psychopathology and behavioral problems. For measures of child cognition, a higher score represented better cognitive ability.

### Statistical analysis

Each score of 39 environmental measures and 30 child characteristics was first standardized with zero mean and unit variance using rank-based inverse Gaussian transformation^33,34^. Pearson’s correlation coefficients were used to explore the associations of individual parental and social environment variables with individual child measures. Bonferroni correction was used to determine the significance of multiple correlations (the number of tests: 1170) at *p* < 0.001.

For multivariate analysis, principal component analyses (PCA) was first performed within all environmental measures and within the child characteristics, respectively^33^. Varimax rotation was applied to factor loadings of the PCs with eigenvalues greater than 1. The component scores were further computed based on the varimax rotated loadings beyond 0.35. This procedure ensured statistical independence of the PCs within the environmental measures and within the child characteristics.

Mixed effect models were used to examine associations of all environmental PCs with each child characteristic PCs. Age, sex and ethnicity were covariates. The information of twins, non-twin siblings, and 21 different research sites was entered as random effects. Bonferroni correction was used to determine the significance of statistical tests (*n* = 48) at *p* < 0.001.

## Results

This study included 8022 out of 11,875 children (mean[SD] age, 9.9 [0.6] years; 47.8% girls; 57.0% white ethnicity) with the complete environmental and child characteristic data. Table 1 lists the 39 environmental measures and 30 child characteristics of subjects with the complete data (*n* = 8002) and all 11,875 subjects. The sample with the complete data (*n* = 8022) did not differ from the whole sample (*n* = 11,875) in most of measures. However, some environmental measures (i.e., household married percentage, parental education, lead risk, and parent report neighborhood safety) and cognitive measures (i.e., picture vocabulary, oral reading, list sorting, card sorting, picture sequencing, RAVLT delayed memory and matrix reasoning) were better in the sample with the complete data than the whole sample (see *p*-values in Table 1). The severity of child psychopathology (i.e., child aggressive behavior, child attention problems, and child rule-breaking behavior from CBCL, and the mania score). was slightly lower in the sample with the complete data than in the whole sample data (see *p*-values in Table 1).

**Table 1:** Demographics, parental and social environmental measures and child outcomes for complete data and full sample data.

	Complete data mean (SD)	Full sample data* mean (SD)	*p*
Age	9.9 (0.6)	9.9 (0.6)	0.954
Gender (%)			0.971
Male	52.2	52.1	
Female	47.8	47.9	
Race/ethinicity (%)			0.000
White	57.0	52.1	
Black	12.2	15.0	
Hispanic	19.0	20.3	
Asian	2.0	2.1	
Other	9.8	10.5	
Parent anxious/depressed	5.0 (4.9)	5.0 (4.9)	0.268
Parent attention problems	4.6 (4.2)	4.6 (4.3)	0.283
Parent aggressive behavior	3.3 (3.5)	3.4 (3.6)	0.086
Parent withdrawn	1.5 (2.0)	1.6 (2.1)	0.007
Parent somatic complaints	2.8 (3.0)	2.9 (3.2)	0.041
Parent thought problems	1.4 (1.7)	1.4 (1.9)	0.010
Parent rule-breaking behavior	1.1 (1.8)	1.2 (1.9)	0.006
Parent intrusive	1.0 (1.4)	1.0 (1.4)	0.759
Family substance use disorder	0.8 (0.8)	0.8 (0.8)	0.480
Family mental disorder	2.5 (2.1)	2.5 (2.1)	0.267
Tobacco use before pregnancy	0.91 (3.3)	1.0 (3.6)	0.011
Tobacco use after pregnancy	0.3 (1.8)	0.3 (2.0)	0.036
Marijuana use before pregnancy	0.1 (0.7)	0.1 (0.7)	0.603
Marijuana use after pregnancy	0.02 (0.24)	0.02 (0.26)	0.505
Alcohol use before pregnancy	0.9 (2.6)	0.9 (2.7)	0.631
Alcohol use after pregnancy	0.04 (0.66)	0.05 (1)	0.175
Pregnancy complications	0.7 (1)	0.7 (1.1)	0.298
Birth complications	0.4 (0.8)	0.4 (0.8)	0.309
Preterm birth	0.9 (2.2)	0.9 (2.2)	0.960
Birth weight (lbs)	6.6 (1.5)	6.6 (1.5)	0.078
Brain injury/concussion	1.1 (0.3)	1.1 (0.3)	0.937
Household married (%)	82.7	80.7	0.000
Parental education (%)			0.000
﻿ <HS diploma	3.5	5.0	
HS diploma/GED	7.8	9.5	
Some college	24.8	26.0	
﻿ Bachelor	27.1	25.4	
Post graduate degree	36.8	34.1	
Household income (%)			0.004
[<50 K]	27.5	29.7	
[≥50 K & < 100 K]	28.6	28.3	
[≥100 K]	43.9	42.1	
Economic insecurity	0.4 (1.0)	0.4 (1.1)	0.002
Area deprivation index	92.0 (25.0)	93.0 (25.0)	0.102
Lead risk	4.9 (3.1)	5.1 (3.1)	0.000
Uniform crime report	49,000 (81,000)	52,000 (85,000)	0.008
Parent report neighborhood safety	3.9 (0.9)	3.9 (1.0)	0.000
Child report neighborhood safety	4.1 (1.1)	4 (1.1)	0.005
School environment	20.0 (2.7)	20.0 (2.8)	0.461
School involvement	13.0 (2.3)	13.0 (2.4)	0.412
School disengagement	3.7 (1.4)	3.7 (1.5)	0.177
Parental monitoring	4.4 (0.5)	4.4 (0.5)	0.038
Parent acceptance	2.8 (0.3)	2.8 (0.3)	0.073
Child report prosocial tendency	1.7 (0.4)	1.7 (0.4)	0.458
Parent report prosocial tendency	1.8 (0.39)	1.8 (0.4)	0.036
Child report (family conflict)	2.0 (1.9)	2.0 (2.0)	0.115
Parent report (family conflict)	2.5 (1.9)	2.5 (2.0)	0.110
Child anxious/depressed	2.5 (3.0)	2.5 (3.1)	0.546
Child thought problems	1.6 (2.1)	1.6 (2.2)	0.073
Child agreesive behavior	3.0 (4.1)	3.3 (4.4)	0.000
Child social problems	1.5 (2.2)	1.6 (2.3)	0.001
Child withdrawn/depressed	1.0 (1.6)	1.0 (1.7)	0.025
Child somatic	1.5 (1.9)	1.5 (2.0)	0.299
Child attention problems	2.8 (3.4)	3 (3.5)	0.000
Child rule-breaking behavior	1.1 (1.7)	1.2 (1.9)	0.000
Mania	1.2 (2.5)	1.3 (2.8)	0.000
Psychosis	5.9 (10.0)	6.3 (11.0)	0.003
Negative urgency	8.4 (2.6)	8.5 (2.6)	0.157
Positive urgency	7.9 (2.9)	8 (3)	0.016
Lack of planning	7.7 (2.4)	7.7 (2.4)	0.906
Lack of perseverance	7.0 (2.2)	7.0 (2.3)	0.139
Sensation seeking	9.8 (2.7)	9.8 (2.7)	0.427
BAS: reward responsiveness	8.8 (2.4)	8.8 (2.4)	0.484
BAS: goal directed behavior	4.0 (3.0)	4.1 (3.1)	0.001
BAS: fun seeking	5.7 (2.6)	5.7 (2.6)	0.386
Behavioral inhibition system	5.5 (2.8)	5.5 (2.8)	0.246
Pciture Vocabulary	85.0 (8.0)	84.0 (8.1)	0.000
Oral reading	91.0 (6.7)	91.0 (6.9)	0.000
List sorting	98.0 (12.0)	97.0 (12.0)	0.000
Flanker	94.0 (8.9)	94.0 (9.1)	0.002
Card sorting	93.0 (9.2)	93.0 (9.5)	0.000
Pattern comparison	88.0 (14.0)	88.0 (15.0)	0.119
Picture sequencing	100.0 (12.0)	100 (12.0)	0.000
LMT accuracy	0.6 (0.2)	0.6 (0.2)	0.003
RAVLT delayed memory	9.3 (3.1)	9.2 (3.2)	0.000
Matrix reasoning	10.0 (2.9)	9.9 (3.0)	0.000
Cash choice	1.6 (0.5)	1.6 (0.5)	0.253

Group differences are tested using two-sample t-test with equal variance assumption for continuous variables and *χ*^2^ tests for discrete variables.

*Due to missing values, the sum of percentages may not equal to 100%.

Figure 1 illustrates significant correlations between 39 parental and social environment measures and 30 child outcomes (Bonferroni corrected *p* < 0.001). This suggested strong correlations between parental and child psychopathology, between socioeconomic status and cognition, between social interactions and child psychopathology, and between proximal social environment and interactions and child impulsive behaviors.

**Fig. 2:**
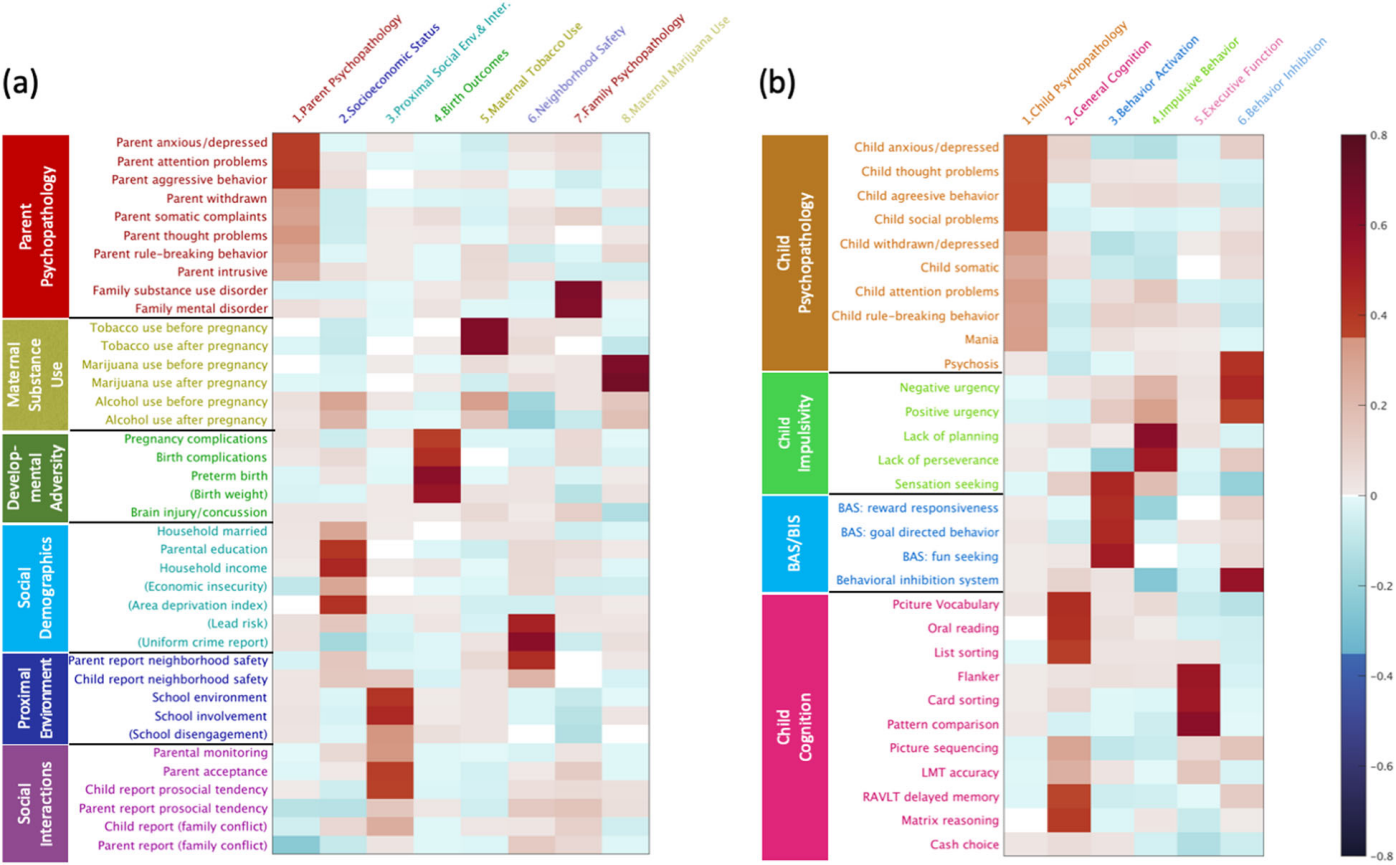
Varimax rotated loadings of retained principal components. **a** Parental, socioeconomic, and social environmental factors have 8 components retained. **b** Child characteristics have 6 components retained. BAS, behavioral activation system; BIS, behavioral inhibition system.

Figure 2 shows 8 PCs for environmental factors (48.7% variance explained) and 6 PCs for child characteristics (51.6% variance explained). The 8 PC environmental factors included (1) *Parent Psychopathology* (14.3% variance explained), *Socioeconomic Status* (7.5% variance explained), (3) *Proximal social environment and interaction* (7.3% variance explained), *Birth Outcomes* (5.1% variance explained), (5) *Maternal Tobacco Use* (4.7% variance explained), (6) *Neighbourhood Safety* (3.5% variance explained), (7) *Family Psychopathology* (3.3% variance explained), and (8) *Maternal Marijuana Use* (3.0% variance explained). The 6 PC child characteristic components included (1) *Child Psychopathology* (17.9% variance explained), *General Cognition* (11.6% variance explained), (3) *Behavioral Activation* (8.3% variance explained), *Impulsive Behavioral Problems* (5.6% variance explained), (5) *Executive Function* (4.3% variance explained), and (6) *Behavioral Inhibition* (3.9% variance explained).

**Fig. 3:**
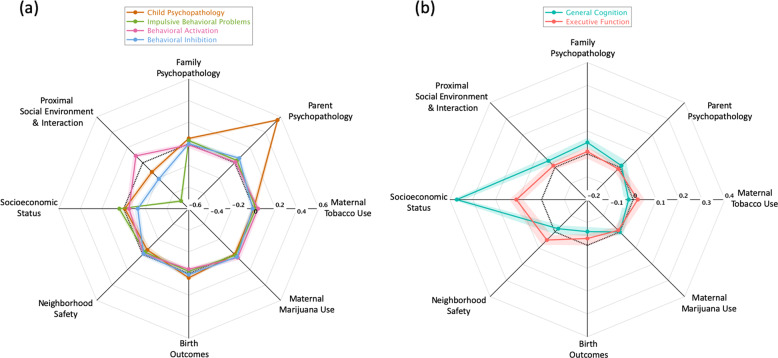
Associations of environmental factors with child characteristics. **a** The standardized regression coefficients of eight parental, socioeconomic, and social environmental components on each child psychopathology and the behavioral components. **b** The standardized regression coefficients of eight parental, socioeconomic, and social environmental components on child general cognition and executive function. In each panel, the colorful rings represent the child characteristic components, and the verteces represent the eight parental, socioeconomic, and social environmental components. From the center to the periphery, the regression coefficients are from negative to positive, and zero is highlighted by the black dash ring. The shade around each colorful ring shows the 95% confidence interval of the corresponding regression coefficient.

After controlling for age, gender, ethnicity, and the other environmental PC factors, greater *Parent Psychopathology* (standardized *β* = 0.56, [0.54, 0.58], *p* < 0.001) was associated with greater *Child Psychopathology* (Fig. 3a). Using available reports on the Teacher Report Form on child behaviors (*n* = 2440), the associations between *Parent Psychopathology* and teacher-reported child behavioral problems remained significant (standardized *β* = 0.06, [0.02, 0.11], *p* = 0.002). Moreover, greater *Socioeconomic Status* was associated with greater child *General Cognition* (standardized *β* = 0.37, [0.34, 0.39], *p* < 0.001) *and Executive Function* (standardized *β* = 0.11, [0.08, 0.14], *p* < 0.001, Fig. 3b) but with less *Behavioral Inhibition* (standardized *β* = −0.13, [−0.16, −0.10], *p* < 0.001; Fig. 3a). Greater *Proximal Social Environment and Interaction* were associated with less child *Impulsive Behavioral Problems* (standardized *β* = −0.50, [−0.52, −0.48], *p* < 0.001) and *Behavioral Inhibition* (standardized *β* = −0.21, [−0.24, −0.19], *p* < 0.001), but greater *Behavioral Activation* (standardized *β* = 0.09, [0.07, 0.12], *p* < 0.001; Fig. 3a). The environmental PCs related to birth outcomes, maternal alcohol, tobacco, and drug use were not significantly related to child psychopathology, behavior, and cognition (Tables 2, 3).

**Table 2:** Associations of all environmental PCs with each child psychopathology and behavior problem PCs.

Variables	Child psychopathology		Behavioral activation		Impulsive behavior		Behavioral inhibition	
	Standardized *β* (95% CI)	*p*	Standardized *β* (95% CI)	*p*	Standardized *β* (95% CI)	*p*	Standardized *β* (95% CI)	*p*
Parent psychopathology	0.56 (0.54 to 0.58)	0.000*	0.01 (-0.02 to 0.03)	0.678	0.03 (0.01 to 0.05)	0.007	0.06 (0.03 to 0.08)	0.000*
Socioeconomic status	−0.01 (−0.04 to 0.01)	0.336	−0.05 (−0.08 to −0.02)	0.001	0.04 (0.01 to 0.06)	0.003	−0.13 (−0.15 to −0.1)	0.000*
Proximal social env. & inter.	−0.12 (−0.14 to −0.1)	0.000*	0.09 (0.07 to 0.12)	0.000*	−0.5 (−0.52 to −0.48)	0.000*	−0.21 (−0.24 to −0.19)	0.000*
Birth outcomes	0.04 (0.02 to 0.06)	0.000*	−0.04 (−0.06 to −0.01)	0.003	−0.02 (−0.04 to 0)	0.089	0.01 (−0.01 to 0.03)	0.387
Maternal tobacco use	0 (−0.02 to 0.02)	0.832	0.04 (0.02 to 0.06)	0.001	0.02 (0 to 0.04)	0.023	−0.01 (−0.03 to 0.02)	0.582
Neighborhood safety	−0.06 (−0.09 to −0.04)	0.000*	−0.01 (−0.04 to 0.01)	0.300	−0.03 (−0.06 to −0.01)	0.010	−0.01 (−0.04 to 0.02)	0.420
Family psychopathology	0.05 (0.03 to 0.06)	0.000*	−0.01 (−0.04 to 0.01)	0.282	0.03 (0.01 to 0.05)	0.006	0 (−0.02 to 0.03)	0.744
Maternal marijuana use	0 (−0.02 to 0.01)	0.598	0.04 (0.02 to 0.07)	0.000^*^	0.01 (−0.01 to 0.03)	0.283	0.03 (0.01 to 0.05)	0.014

*The significant results with Bonferroni corrected *p* < 0.01.

**Table 3:** Associations of all environmental PCs with each child cognitive PCs.

Variables	General cognition		Executive function	
	Standardized *β* (95% CI)	*p*	Standardized *β* (95% CI)	*p*
Parent psychopathology	0.01 (−0.01 to 0.04)	0.164	−0.01 (−0.03 to 0.01)	0.434
Socioeconomic status	0.37 (0.34 to 0.39)	0.000*	0.11 (0.08 to 0.14)	0.000*
Proximal social env. & inter.	0.04 (0.02 to 0.06)	0.000*	0.01 (−0.01 to 0.03)	0.355
Birth outcomes	−0.06 (−0.08 to −0.04)	0.000*	−0.03 (−0.05 to 0)	0.025
Maternal tobacco use	−0.02 (−0.04 to 0)	0.021	0.02 (0 to 0.05)	0.051
Neighborhood safety	−0.02 (−0.04 to 0.01)	0.199	0.05 (0.02 to 0.08)	0.001
Family psychopathology	0.05 (0.03 to 0.07)	0.000*	0.01 (−0.02 to 0.03)	0.546
Maternal marijuana use	0.00 (−0.02 to 0.02)	0.977	−0.01 (−0.03 to 0.01)	0.349

*The significant results with Bonferroni corrected *p* < 0.01.

Our repeated analyses using the full study sample (*n* = 11,875) and mean imputation for missingness showed the similar findings as stated above (in Supplementary Figs. S1 and S2 of the Supplementary Material).

## Discussion

This study showed the distinctive influences of the parental, socioeconomic, and social environmental factors on child psychopathology, behavioral problems, and cognition. As expected, strong relationships were found between *Parent Psychopathology* and *Child Psychopathology*, between *Socioeconomic Status* and child *Cognition*, and between *Proximal Social Environment and Interaction* and child *Impulsive behaviors*. What was unexpected, however, was our lack of identifying relationships between birth outcomes, maternal tobacco and drug use with child psychopathology, behavioral problems, and cognition.

Consistent with previous findings^35,36^, we found strong association between the psychopathology in parents and their children. Child psychopathology was assessed by parents and thus there is a tendency that parents with greater psychopathology will also rate their child as having greater psychopathology. When we utilized teacher reported behavioral problems of the child and parent self-report, the association remained significant, albeit less strong. Our findings provide further support for a potential genetic contribution for the transgenerational transmission of psychopathology from parents to behavioral characteristics of children.

This study also identified the associations of *Socioeconomic Status* with child *General Cognition* and *Executive Function*. This is congruent with previous findings, suggesting that lower *Socioeconomic Status* strongly predicts lower IQ and executive functions^3,37^. Most of previous studies employ household income and/or parental education or both as the representation of *Socioeconomic Status*^20^. In contrast, we quantified *Socioeconomic Status* using a broad construct that incorporated variation not only from household income and parental education, but also from a regional deprivation index. From this aspect, our study provided evidence supporting the idea of a reduction of poverty and increasing education at the level of both family and neighbourhood may help improve child cognitive development.

Unlike previous studies^38,39^, our findings did not support strong associations of birth outcomes, maternal tobacco, and drug use with child psychopathology, behavior, and cognition in this general child population. Nevertheless, when analyzing the association between maternal marijuana use and psychosis, we showed the similar result (*p* = 0.014 in Table 2) as that presented in^15^. The lack of such associations among the PC scores is partly because our findings were obtained after controlling for *Parental Psychopathology*, *Socioeconomic Status*, and etc, suggesting that *Parental Psychopathology* and *Socioeconomic Status* had a greater effect on child neurodevelopmental outcomes. Most of existing studies generally focus only on a case-control or imbalanced designs and do not assess the comprehensive profile of parental, socioeconomic, and social factors and hence may not quantify true effects of maternal tobacco and drug use as well as birth outcomes on child neurodevelopment in a general population^40^.

One of the strengths of our study is that we employed a large population-based sample of children who are all participating in the ABCD baseline wave of data collection. Thus, we were able to incorporate a comprehensive assessment of parental, socioeconomic, and social environmental factors as well as child characteristics. Nevertheless, the reliance on cross-sectional data precludes any determination of causality. Moreover, the ABCD study sampled from the United States, which may limit the generalizability of our findings. Further research is necessary to explore across other ethnicities and cultures to enhance the potential generalization of our findings.

Our findings suggest that parental psychopathology, socioeconomic status, and social environment and interactions are the strongest risks for behavioral problems and cognitive performance in a general child population. These children should be targeted for intervention programs, with the possibility for including both primary and secondary prevention.

## Supplementary information

Supplementary Material

## References

[1] Wang, Q. et al. Sex-dependent associations among maternal depressive dymptoms, child reward network, and behaviors in early childhood. *Cereb. Cortex*10.1093/cercor/bhz135 (2019). 10.1093/cercor/bhz135 31339998

[2] Clark CA, Woodward LJ, Horwood LJ, Moor S (2008). Development of emotional and behavioral regulation in children born extremely preterm and very preterm: biological and social influences. Child Dev..

[3] Noble KG (2015). Family income, parental education and brain structure in children and adolescents. Nat. Neurosci..

[4] Almas AN (2020). The impact of caregiving disruptions of previously institutionalized children on multiple outcomes in late childhood. Child Dev..

[5] Potijk MR, Kerstjens JM, Bos AF, Reijneveld SA, de Winter AF (2013). Developmental delay in moderately preterm-born children with low socioeconomic status: risks multiply. J. Pediatr..

[6] Harnish JD, Dodge KA, Valente E (1995). Mother-child interaction quality as a partial mediator of the roles of maternal depressive symptomatology and socioeconomic status in the development of child behavior problems. Conduct problems prevention research group. Child Dev..

[7] Garavan H (2018). Recruiting the ABCD sample: design considerations and procedures. Dev. Cogn. Neurosci..

[8] Barch DM (2018). Demographic, physical and mental health assessments in the adolescent brain and cognitive development study: Rationale and description. Dev. Cogn. Neurosci..

[9] Clark DB (2018). Biomedical ethics and clinical oversight in multisite observational neuroimaging studies with children and adolescents: The ABCD experience. Dev. Cogn. Neurosci..

[10] Zucker RA (2018). Assessment of culture and environment in the Adolescent Brain and Cognitive Development Study: Rationale, description of measures, and early data. Dev. Cogn. Neurosci..

[11] Achenbach, T. Achenbach System of Empirically Based Assessment (ASEBA): Development, Findings, Theory, and Applications. *Encyclopedia of Autism Spectrum Disorders*.

[12] Rice JP (1995). Comparison of direct interview and family history diagnoses of alcohol dependence. Alcohol. Clin. Exp. Res..

[13] Kessler RC (2009). Design and field procedures in the US National Comorbidity Survey Replication Adolescent Supplement (NCS-A). Int. J. Methods Psychiatr. Res..

[14] Kessler RC (2009). National comorbidity survey replication adolescent supplement (NCS-A): II. Overview and design. J. Am. Acad. Child Adolesc. Psychiatry.

[15] Fine, J. D. et al. Association of prenatal cannabis exposure with psychosis proneness among children in the Adolescent Brain Cognitive Development (ABCD) Study. *JAMA Psychiatry* 4–6 10.1001/jamapsychiatry.2019.0076 (2019). 10.1001/jamapsychiatry.2019.0076 PMC6583849 30916716

[16] Bogner JA (2017). Test-retest reliability of traumatic brain injury outcome measures. J. Head. Trauma Rehabil..

[17] Hamilton CM (2011). The PhenX Toolkit: get the most from your measures. Am. J. Epidemiol..

[18] Diemer MA, Mistry RS, Wadsworth ME, López I, Reimers F (2013). Best practices in conceptualizing and measuring social class in psychological research. Anal. Soc. Issues Public Policy.

[19] Kind AJH (2014). Neighborhood socioeconomic disadvantage and 30-day rehospitalization. Ann. Intern. Med..

[20] Farah MJ (2017). The neuroscience of socioeconomic status: correlates, causes, and consequences. Neuron.

[21] Echeverria SE (2004). Reliability of self-reported neighborhood characteristics. J. Urban Heal. Bull. N. Y. Acad. Med..

[22] Mujahid MS, Diez Roux AV, Morenoff JD, Raghunathan T (2007). Assessing the measurement properties of neighborhood scales: from psychometrics to ecometrics. Am. J. Epidemiol..

[23] Stover PJ, Harlan WR, Hammond JA, Hendershot T, Hamilton CM (2010). PhenX: A toolkit for interdisciplinary genetics research. Curr. Opin. Lipidol..

[24] Chilcoat HD, Anthony JC (1996). Impact of parent monitoring on initiation of drug use through late childhood. J. Am. Acad. Child Adolesc. Psychiatry.

[25] Schaefer ES (1965). A configurational analysis of children’s reports of parent behavior. J. Consult. Psychol..

[26] Goodman R, Meltzer H, Bailey V (2003). The strengths and difficulties questionnaire: a pilot study on the validity of the self-report version. Int. Rev. Psychiatry.

[27] Moos, R. H. & Moos, B. S. *Family Environment Scale manual*. (Consulting Psychologists Press, Palo Alto, CA, 1986).

[28] Luciana M (2018). Adolescent neurocognitive development and impacts of substance use: Overview of the adolescent brain cognitive development (ABCD) baseline neurocognition battery. Dev. Cogn. Neurosci..

[29] Youngstrom EA, Frazier TW, Demeter C, Calabrese JR, Findling RL (2008). Developing a 10-Item mania scale from the parent general behavior inventory for children and adolescents. J. Clin. Psychiatry.

[30] Loewy RL, Therman S, Manninen M, Huttunen MO, Cannon TD (2012). Prodromal psychosis screening in adolescent psychiatry clinics. Early Interv. Psychiatry.

[31] Zapolski TCB, Stairs AM, Settles RF, Combs JL, Smith GT (2010). The measurement of dispositions to rash action in children. Assessment.

[32] Carver CS, White TL (1994). Behavioral inhibition, behavioral activation, and affective responses to impending reward and punishment: The BIS/BAS Scales. J. Pers. Soc. Psychol..

[33] Thompson WK (2019). The structure of cognition in 9 and 10 year-old children and associations with problem behaviors: Findings from the ABCD study’s baseline neurocognitive battery. Dev. Cogn. Neurosci..

[34] Miller KL (2016). Multimodal population brain imaging in the UK Biobank prospective epidemiological study. Nat. Neurosci..

[35] Befera Mschulte, Barkley RA (1985). Hyperactive and normal girls and boys: mother-child interaction, parent psychiatric status and child psychopathology. J. Child Psychol. Psychiatry.

[36] Gunlicks ML, Weissman MM (2008). Change in child psychopathology with improvement in parental depression: a systematic review. J. Am. Acad. Child Adolesc. Psychiatry.

[37] Turkheimer E, Haley A, Waldron M, D’Onofrio B, Gottesman II (2003). Socioeconomic status modifies heritability of IQ in young children. Psychol. Sci..

[38] Bhutta, A. T., Cleves, M. A., Casey, P. H., Cradock, M. M. & Anand, K. J. S. Cognitive and behavioral outcomes of school-aged children who were born preterm. *JAMA* **288**, 728 (2002). 10.1001/jama.288.6.728 12169077

[39] Huizink AC, Mulder EJH (2006). Maternal smoking, drinking or cannabis use during pregnancy and neurobehavioral and cognitive functioning in human offspring. Neurosci. Biobehav. Rev..

[40] Huizink AC (2014). Prenatal cannabis exposure and infant outcomes: overview of studies. Prog. Neuro-Psychopharmacol. Biol. Psychiatry.

