# Supplementary material for: Parental and social factors in relation to child psychopathology, behavior, and cognitive function
Source: Transl Psychiatry. 2020 Feb 26;10:80. doi: 10.1038/s41398-020-0761-6 (PMC7044210; doi:10.1038/s41398-020-0761-6)
Supplement: Supplementary file 1 — Supplementary Material [file 41398_2020_761_MOESM1_ESM.docx]

**Supplementary Materials**

**
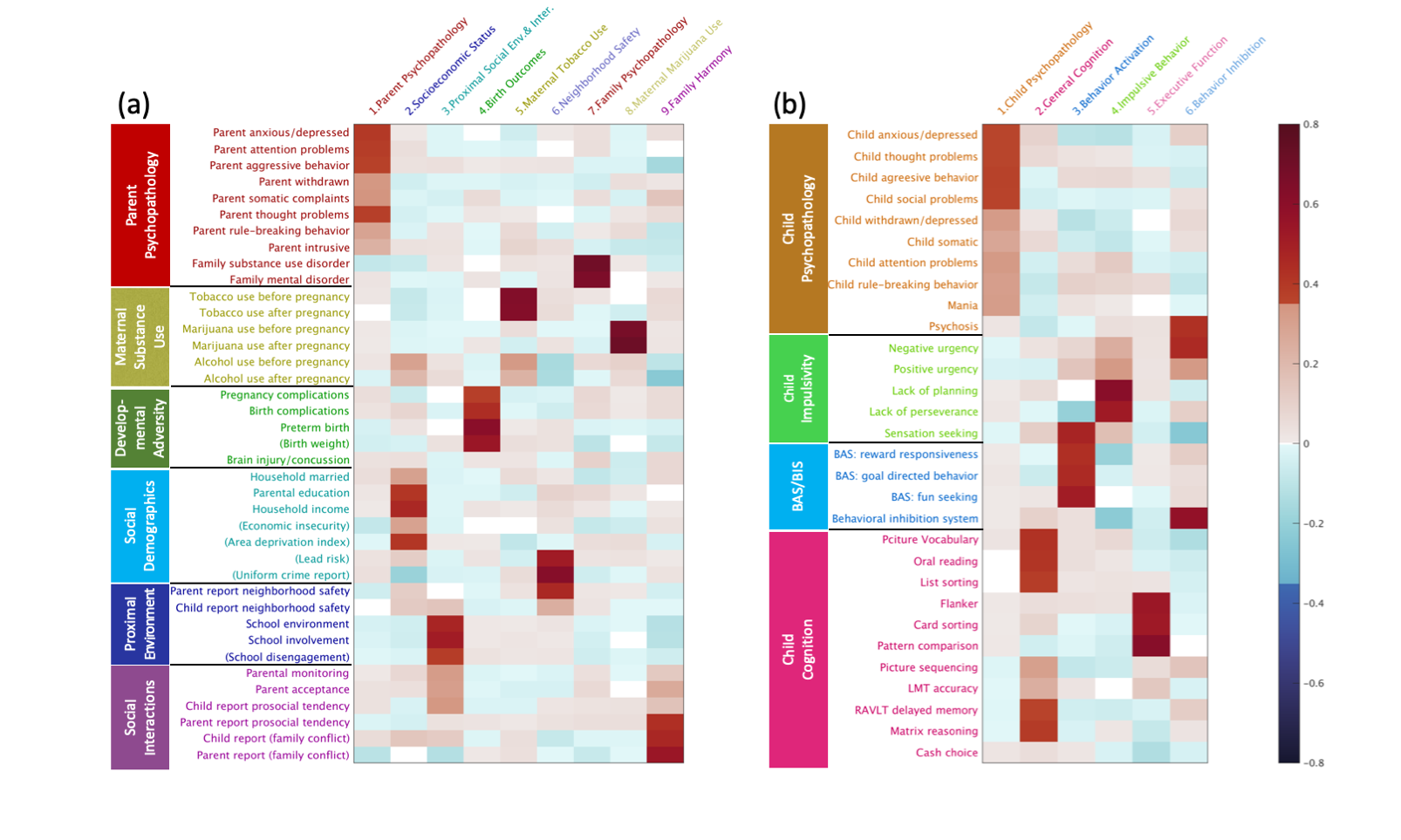
**

**Figure S1. Varimax rotated loadings of retained principal components using the full study sample (n=11,875).** **Panel (a)** parental, socioeconomic, and social environmental factors have 9 components retained which is similar to findings using the complete sample but shows an additional component Family Harmony. **Panel (b)** child characteristics have 6 components retained which is also similar to findings using the complete sample.

Abbreviations: BAS, behavioral activation system; BIS, behavioral inhibition system.

**
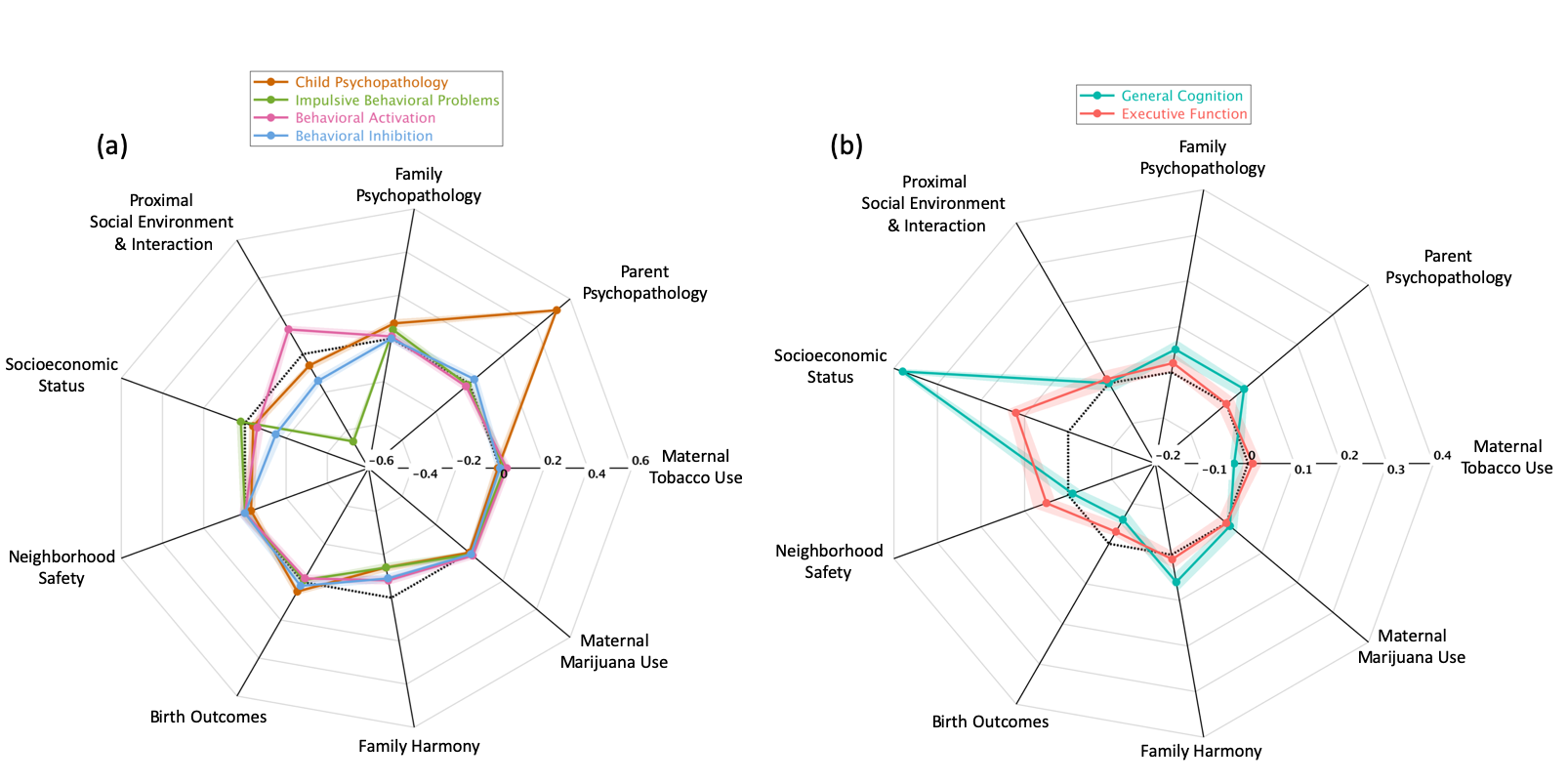
**

**Figure S2. Associations of environmental factors with child characteristics using the full study sample (n=11,875).** **Panel (a)** shows the standardized regression coefficients of nine parental, socioeconomic, and social environmental components on each child psychopathology and the behavioral components. **Panel (b)** shows the standardized regression coefficients of nine parental, socioeconomic, and social environmental components on child general cognition and executive function. In each panel, the colorful rings represent the child characteristic components, and the verteces represent the eight parental, socioeconomic, and social environmental components. From the center to the periphery, the regression coefficients are from negative to positive, and zero is highlighted by the black dash ring. The shade around each colorful ring shows the 95% confidence interval of the corresponding regression coefficient.

**Table S1.** The NDAR Global Unique Identifier (GUID) of subjects with missing values. The data from these subjects were not included.

| NDAR_INV003RTV85 | NDAR_INV5784LZWF | NDAR_INVAKCU5UPL | NDAR_INVFZ7AMNW3 | NDAR_INVMC7UEVZ1 | NDAR_INVULRUN522 |
| --- | --- | --- | --- | --- | --- |
| NDAR_INV005V6D2C | NDAR_INV578DB0G5 | NDAR_INVAKG9Z7UN | NDAR_INVFZ7T2G2T | NDAR_INVMCTA6E68 | NDAR_INVULY80GEF |
| NDAR_INV00HEV6HB | NDAR_INV57TXVRYZ | NDAR_INVAKLPG0GB | NDAR_INVFZ8BBNJK | NDAR_INVMCYKEL17 | NDAR_INVULZMADF1 |
| NDAR_INV00J52GPG | NDAR_INV589PDMA0 | NDAR_INVAKPAUM7W | NDAR_INVFZC8ZGMR | NDAR_INVMD3JLFR5 | NDAR_INVUM9EFLC3 |
| NDAR_INV0182J779 | NDAR_INV58G3ZX3W | NDAR_INVAL8NAKXY | NDAR_INVFZCKB9W4 | NDAR_INVMDF3BYV1 | NDAR_INVUMHGTLW8 |
| NDAR_INV01D03VR7 | NDAR_INV58P73L9E | NDAR_INVALAAC3EU | NDAR_INVFZWKRBW7 | NDAR_INVMEGDWWCW | NDAR_INVUN1M9EZ6 |
| NDAR_INV01ELX9L6 | NDAR_INV59BE4FA2 | NDAR_INVALKAWEWB | NDAR_INVG026P1C9 | NDAR_INVMEZY36AN | NDAR_INVUN26G8WH |
| NDAR_INV01EN91PG | NDAR_INV59C91GGB | NDAR_INVALWW1VUJ | NDAR_INVG04AEYYX | NDAR_INVMF00X6VN | NDAR_INVUN2A7AA5 |
| NDAR_INV029PWCFY | NDAR_INV59MF6Y4H | NDAR_INVAM8K670H | NDAR_INVG08VWT16 | NDAR_INVMF0LLWMJ | NDAR_INVUNWBU521 |
| NDAR_INV030W95VP | NDAR_INV59MJGL9R | NDAR_INVAMLLW49U | NDAR_INVG0F3TJPW | NDAR_INVMF6NM996 | NDAR_INVUP7H8Z59 |
| NDAR_INV03BDCNWM | NDAR_INV59W523AL | NDAR_INVAN03AH80 | NDAR_INVG0MXBE3C | NDAR_INVMG9REUL8 | NDAR_INVUPDUETFG |
| NDAR_INV03CV8RDT | NDAR_INV5A4EYCUX | NDAR_INVAN1G06D2 | NDAR_INVG0XX710G | NDAR_INVMGH9KKCV | NDAR_INVUPKM7CMH |
| NDAR_INV03NW0RKL | NDAR_INV5AEUN2YL | NDAR_INVAN3D7PHM | NDAR_INVG13L2THF | NDAR_INVMGPDYH6P | NDAR_INVUPPU9A5W |
| NDAR_INV04BFG4JM | NDAR_INV5AX7X2LE | NDAR_INVANDMZJM4 | NDAR_INVG16RFRXX | NDAR_INVMHCA4296 | NDAR_INVUR7GBKPR |
| NDAR_INV04CLBZAD | NDAR_INV5B0XG3JG | NDAR_INVANEX1Y2H | NDAR_INVG1CR55JE | NDAR_INVMHPNB2Y1 | NDAR_INVURFDN3WC |
| NDAR_INV04P0G6LK | NDAR_INV5B5DML2V | NDAR_INVANMZGAVD | NDAR_INVG1TXFJX2 | NDAR_INVMHR6ZFBJ | NDAR_INVURG8199G |
| NDAR_INV04TRXUGL | NDAR_INV5BXCGPHW | NDAR_INVAP0P9PX1 | NDAR_INVG1U31EWC | NDAR_INVMJC3XJ60 | NDAR_INVURJ3XK7W |
| NDAR_INV052HU3CU | NDAR_INV5CAAJ5TK | NDAR_INVAP80JDC6 | NDAR_INVG25VMDZ4 | NDAR_INVMJH6HGA1 | NDAR_INVURNAH8GZ |
| NDAR_INV059VLAHF | NDAR_INV5D540DWZ | NDAR_INVAPWWNB9N | NDAR_INVG270KBWC | NDAR_INVMJMFXLDG | NDAR_INVURX0F423 |
| NDAR_INV05WK8AN7 | NDAR_INV5DJB8FFX | NDAR_INVAPYPHXZ7 | NDAR_INVG2K6ZE55 | NDAR_INVMJPLXBJT | NDAR_INVUTBRXV0G |
| NDAR_INV06A9DLE9 | NDAR_INV5DNKJ4H4 | NDAR_INVARJG7LG1 | NDAR_INVG2P3W364 | NDAR_INVMK65MX3T | NDAR_INVUTCRUX6L |
| NDAR_INV06DE9Y0L | NDAR_INV5DW63RTW | NDAR_INVARKPPU88 | NDAR_INVG2PGTD44 | NDAR_INVMKNER6N9 | NDAR_INVUTEVFWND |
| NDAR_INV06DJHEV0 | NDAR_INV5E97AP49 | NDAR_INVARVF2D21 | NDAR_INVG31B74F8 | NDAR_INVMKPVUMW3 | NDAR_INVUTVE98D5 |
| NDAR_INV06U4DYFY | NDAR_INV5EFUXC2G | NDAR_INVAT1UAHM3 | NDAR_INVG34U0PZU | NDAR_INVMKRG2K7G | NDAR_INVUU44L176 |
| NDAR_INV06WHVUPX | NDAR_INV5F2R3TL8 | NDAR_INVATE29B31 | NDAR_INVG3RUAU07 | NDAR_INVMKZM84DW | NDAR_INVUULNDK83 |
| NDAR_INV07CMT243 | NDAR_INV5F54ZMX4 | NDAR_INVATZ3RNRJ | NDAR_INVG47HFECU | NDAR_INVML43DJ88 | NDAR_INVUURWXJBP |
| NDAR_INV07RAHHYH | NDAR_INV5FB3U0EJ | NDAR_INVAU8YJ8BH | NDAR_INVG48P9551 | NDAR_INVML6ZF1HE | NDAR_INVUV48BM08 |
| NDAR_INV07THBLHG | NDAR_INV5FB99JMV | NDAR_INVAUGA1ZYM | NDAR_INVG5PC310E | NDAR_INVMLDY2ZT2 | NDAR_INVUVKK59DF |
| NDAR_INV086114AT | NDAR_INV5FUBUX54 | NDAR_INVAUK18A9C | NDAR_INVG5XLB72Y | NDAR_INVMLFXUXUL | NDAR_INVUVXX7NXJ |
| NDAR_INV0889M0JE | NDAR_INV5G3DP835 | NDAR_INVAUR9UZH4 | NDAR_INVG66DDVRA | NDAR_INVMLU3DJN0 | NDAR_INVUW2RRYUA |
| NDAR_INV08DNLREC | NDAR_INV5G46RBZN | NDAR_INVAUW9X5KJ | NDAR_INVG6JBZCCD | NDAR_INVMM0GMFCC | NDAR_INVUW7TPCMK |
| NDAR_INV08FUB58A | NDAR_INV5GAREWU2 | NDAR_INVAV0XTVFA | NDAR_INVG7BRDFU8 | NDAR_INVMM3Y00ZD | NDAR_INVUWH8CG0A |
| NDAR_INV08K8VF33 | NDAR_INV5HG40TVB | NDAR_INVAV39CN05 | NDAR_INVG7DT8J7N | NDAR_INVMM5Z9B33 | NDAR_INVUWH8GXXW |
| NDAR_INV08P1JKNE | NDAR_INV5HKYD59J | NDAR_INVAV4XUTD7 | NDAR_INVG7UNU2FK | NDAR_INVMMLVDCJH | NDAR_INVUWLUMMVY |
| NDAR_INV08P3GNDV | NDAR_INV5HNAA4NT | NDAR_INVAVEFGTH2 | NDAR_INVG7XE917X | NDAR_INVMMPNAM6J | NDAR_INVUWNVH2C7 |
| NDAR_INV095EVLDD | NDAR_INV5J1VTUME | NDAR_INVAVX9HYUA | NDAR_INVG8990TE7 | NDAR_INVMMY60CD5 | NDAR_INVUWTWYGD1 |
| NDAR_INV09AEBLZH | NDAR_INV5KGZR02X | NDAR_INVAW1XZUW5 | NDAR_INVG8B9LC6Y | NDAR_INVMN7K3JN0 | NDAR_INVUX20B6L5 |
| NDAR_INV09C3ZNFZ | NDAR_INV5L9NEM2D | NDAR_INVAWA5076Z | NDAR_INVG8VWC1TN | NDAR_INVMP6JFH4L | NDAR_INVUXT483FW |
| NDAR_INV09VBPCLU | NDAR_INV5LM4TFDU | NDAR_INVAWAEU3P4 | NDAR_INVG91T2JF8 | NDAR_INVMPD4KFF5 | NDAR_INVUXXJ24L8 |
| NDAR_INV09ZE6UUK | NDAR_INV5MF8VMU6 | NDAR_INVAWG2NZC4 | NDAR_INVG957G5YY | NDAR_INVMPPV8539 | NDAR_INVUY1UGEA3 |
| NDAR_INV0A86UD86 | NDAR_INV5MHT9RR4 | NDAR_INVAWMVEC49 | NDAR_INVG9MENY7V | NDAR_INVMPZ9B1D7 | NDAR_INVUY8GTG7G |
| NDAR_INV0A87RKWD | NDAR_INV5MJHVEB8 | NDAR_INVAWNDT22T | NDAR_INVG9U18UHH | NDAR_INVMR60CAWU | NDAR_INVUYU4JRJA |
| NDAR_INV0AEBMADL | NDAR_INV5MP2XXGP | NDAR_INVAWP77RE3 | NDAR_INVG9W26JX4 | NDAR_INVMTHF6N0P | NDAR_INVUZ89A2LM |
| NDAR_INV0AU5R8NA | NDAR_INV5MXTGY8D | NDAR_INVAWYGG9MN | NDAR_INVGA8XJ9W5 | NDAR_INVMTJ5Z63Z | NDAR_INVV016WX7A |
| NDAR_INV0B0HVEYX | NDAR_INV5MYF77YV | NDAR_INVAXXG3HHV | NDAR_INVGAFB8D70 | NDAR_INVMTJ6URWD | NDAR_INVV05P2AW1 |
| NDAR_INV0B3PHZ2W | NDAR_INV5N1N1DEL | NDAR_INVAY9W0F99 | NDAR_INVGALZWY31 | NDAR_INVMTJLG5JL | NDAR_INVV0BGPHNW |
| NDAR_INV0BKE31EY | NDAR_INV5N8A69PF | NDAR_INVAYAW3D7X | NDAR_INVGAYGT0LJ | NDAR_INVMTVMYZRH | NDAR_INVV0E701P6 |
| NDAR_INV0C765WK4 | NDAR_INV5NPRNJAU | NDAR_INVAYCZM73P | NDAR_INVGB1LBZ5W | NDAR_INVMU5A4CM4 | NDAR_INVV0UVZ6YT |
| NDAR_INV0CCEN5K2 | NDAR_INV5NVYVN9F | NDAR_INVAYDXW667 | NDAR_INVGB26JEH6 | NDAR_INVMUG529LW | NDAR_INVV104HFJY |
| NDAR_INV0CCMBWPE | NDAR_INV5NZW4ZBJ | NDAR_INVAYLJ9DAH | NDAR_INVGB95PJTK | NDAR_INVMUJR2Y8Y | NDAR_INVV18LRYXU |
| NDAR_INV0CFR33F0 | NDAR_INV5P2UAGPW | NDAR_INVAZ1GVXNZ | NDAR_INVGB9TAJZ8 | NDAR_INVMVEW4R8F | NDAR_INVV1UUN23J |
| NDAR_INV0CV2Y4YR | NDAR_INV5P5E90FX | NDAR_INVAZEURR81 | NDAR_INVGBLP7T9X | NDAR_INVMVEZ4E20 | NDAR_INVV1VLC8FR |
| NDAR_INV0D5J9T8P | NDAR_INV5PTVY6KC | NDAR_INVB0DK62E0 | NDAR_INVGBTLTG2G | NDAR_INVMVPDD8ZG | NDAR_INVV1W73MWR |
| NDAR_INV0DWBRWHT | NDAR_INV5R386XD4 | NDAR_INVB0G69H1H | NDAR_INVGBTXFJC6 | NDAR_INVMVTF3GGD | NDAR_INVV1XY0F8C |
| NDAR_INV0DYF4WPG | NDAR_INV5RY4ZHHG | NDAR_INVB0M8PHVN | NDAR_INVGBUMHM8K | NDAR_INVMW3NKY5F | NDAR_INVV1Y99ECP |
| NDAR_INV0E9UFT0K | NDAR_INV5T6VPW2C | NDAR_INVB15ECXNF | NDAR_INVGC4RRGD2 | NDAR_INVMWC4BV86 | NDAR_INVV1Z1XF3X |
| NDAR_INV0EXY6KFW | NDAR_INV5T9K540P | NDAR_INVB18N983V | NDAR_INVGC7NCM6H | NDAR_INVMWM6VBUW | NDAR_INVV2R0HB8H |
| NDAR_INV0FAET6YA | NDAR_INV5TB3M4RT | NDAR_INVB1DNH7JC | NDAR_INVGCDY2UAK | NDAR_INVMWTU519G | NDAR_INVV2TL3DA9 |
| NDAR_INV0FUC15ZB | NDAR_INV5TEDV177 | NDAR_INVB1EW737N | NDAR_INVGCL0XR2C | NDAR_INVMWTUT7TZ | NDAR_INVV2Y2RFVK |
| NDAR_INV0G42TBW1 | NDAR_INV5TJZTXMY | NDAR_INVB228FCJ6 | NDAR_INVGD1EZA7B | NDAR_INVMX57RL1Z | NDAR_INVV37537GK |
| NDAR_INV0G9MD8JA | NDAR_INV5TT0JL6Z | NDAR_INVB2FVBDTD | NDAR_INVGD45AN4V | NDAR_INVMXGF4JT5 | NDAR_INVV3Z0YVUT |
| NDAR_INV0GGJJ4H9 | NDAR_INV5TT4VN2V | NDAR_INVB2PT115G | NDAR_INVGDJR5U4A | NDAR_INVMXGV952X | NDAR_INVV49BV0FE |
| NDAR_INV0GUTM6AM | NDAR_INV5U642ALM | NDAR_INVB2ZN28L8 | NDAR_INVGDX7FYYH | NDAR_INVMXNTYPJU | NDAR_INVV4XY140Z |
| NDAR_INV0GVW93X6 | NDAR_INV5U8RGRGL | NDAR_INVB49HX84F | NDAR_INVGE15ZEP5 | NDAR_INVMXZ3JJ2G | NDAR_INVV53R103B |
| NDAR_INV0GYC1NWR | NDAR_INV5U9JTZ3X | NDAR_INVB4CA1YNH | NDAR_INVGE320R1H | NDAR_INVMY3YJ5RU | NDAR_INVV54EMBM7 |
| NDAR_INV0GZM9UZJ | NDAR_INV5UAWUVFC | NDAR_INVB4XP9VV8 | NDAR_INVGEBYNHG3 | NDAR_INVMY85AAAM | NDAR_INVV5BGK5FU |
| NDAR_INV0GZN1747 | NDAR_INV5UEM3B36 | NDAR_INVB4Z46M1B | NDAR_INVGFB2AURV | NDAR_INVMYECMVTU | NDAR_INVV5WEC3Y1 |
| NDAR_INV0HB853U9 | NDAR_INV5UHHRZBV | NDAR_INVB5VJ3KEG | NDAR_INVGFDT73J0 | NDAR_INVMYKKZ4YM | NDAR_INVV5Y81HT9 |
| NDAR_INV0J2UAG61 | NDAR_INV5UJ0L2AV | NDAR_INVB60GTH59 | NDAR_INVGFGFUH9T | NDAR_INVMYUP1YJ7 | NDAR_INVV5YL6694 |
| NDAR_INV0J6UAD2Y | NDAR_INV5UYTUJ33 | NDAR_INVB68067CD | NDAR_INVGFH7TNNH | NDAR_INVMZG6CUGJ | NDAR_INVV6822VJD |
| NDAR_INV0J9U5XRJ | NDAR_INV5VAD4B1H | NDAR_INVB6AN7YDK | NDAR_INVGG6CEFZP | NDAR_INVMZKNKER7 | NDAR_INVV6CX3VRR |
| NDAR_INV0JCWR24P | NDAR_INV5VETDCUY | NDAR_INVB6R2TDV5 | NDAR_INVGG87HRBT | NDAR_INVMZYRHT83 | NDAR_INVV6PCNENZ |
| NDAR_INV0JR6Y529 | NDAR_INV5VGKMHCR | NDAR_INVB70M4Z6D | NDAR_INVGGJJVCTG | NDAR_INVN08WN4MR | NDAR_INVV6XHZB59 |
| NDAR_INV0JRNB4U4 | NDAR_INV5VLNFBN7 | NDAR_INVB72KCUK5 | NDAR_INVGGTCPFBU | NDAR_INVN0AK70MR | NDAR_INVV77F4ARK |
| NDAR_INV0JW2EU4V | NDAR_INV5VP4RK9P | NDAR_INVB7BG5TYR | NDAR_INVGGZ80H14 | NDAR_INVN0R3YY02 | NDAR_INVV7EUYJMM |
| NDAR_INV0KTEM27A | NDAR_INV5VRVMVVT | NDAR_INVB7CUAZH9 | NDAR_INVGH70J8FC | NDAR_INVN0XCHU07 | NDAR_INVV7Z95HZ1 |
| NDAR_INV0L1GB53L | NDAR_INV5VWTWJ9Y | NDAR_INVB7DEY5F6 | NDAR_INVGH9BLP1B | NDAR_INVN19DGR09 | NDAR_INVV8576DMV |
| NDAR_INV0L974J49 | NDAR_INV5WBZDXME | NDAR_INVB7FUD8ZP | NDAR_INVGHKZJAH9 | NDAR_INVN1HATRZN | NDAR_INVV874VP8D |
| NDAR_INV0LA6MBBY | NDAR_INV5WE268D9 | NDAR_INVB8J23RH6 | NDAR_INVGJ465HAT | NDAR_INVN1J48BYV | NDAR_INVV8VK6RGX |
| NDAR_INV0LN1KD13 | NDAR_INV5WFFJBKU | NDAR_INVB8N5TFHV | NDAR_INVGJFEZAEG | NDAR_INVN1TKYKW1 | NDAR_INVV96G6YFB |
| NDAR_INV0LXYGL0Y | NDAR_INV5WPTZ0W9 | NDAR_INVB9G2X39Y | NDAR_INVGJPM6GV9 | NDAR_INVN1UYXP41 | NDAR_INVV9D0WWZM |
| NDAR_INV0MPBK7TU | NDAR_INV5WTABLWY | NDAR_INVB9UHUNKZ | NDAR_INVGK2BKPLT | NDAR_INVN1Y93M5A | NDAR_INVV9G24V48 |
| NDAR_INV0MXJ7AZN | NDAR_INV5X8TYY0W | NDAR_INVBAATCKCB | NDAR_INVGKARWYND | NDAR_INVN29F40LG | NDAR_INVV9RZ5E55 |
| NDAR_INV0N10CEEL | NDAR_INV5XAZTBUE | NDAR_INVBB0FDDL0 | NDAR_INVGKUP6C90 | NDAR_INVN2CM6UDZ | NDAR_INVVA90J10J |
| NDAR_INV0NGG5VLJ | NDAR_INV5XPN7DR4 | NDAR_INVBB8UACMP | NDAR_INVGKYBATFU | NDAR_INVN2CVFJPN | NDAR_INVVAMT0KW4 |
| NDAR_INV0NNE55L0 | NDAR_INV5XRG1L4T | NDAR_INVBB9X243N | NDAR_INVGL3ZGV38 | NDAR_INVN2H3FKB7 | NDAR_INVVAPJ5TBV |
| NDAR_INV0P0GTDY0 | NDAR_INV5XT9TNDL | NDAR_INVBBE2GWRH | NDAR_INVGLNY79U6 | NDAR_INVN2MD051W | NDAR_INVVAXL08MG |
| NDAR_INV0P4FUMM3 | NDAR_INV5YHWBYRD | NDAR_INVBC53RFDD | NDAR_INVGLRAHCZN | NDAR_INVN2R08D31 | NDAR_INVVB04BHVZ |
| NDAR_INV0P4XZMZA | NDAR_INV5YKAB0PN | NDAR_INVBCEBWGDG | NDAR_INVGLRTPTNN | NDAR_INVN2RB0FR4 | NDAR_INVVB0K1CTL |
| NDAR_INV0PHTY15N | NDAR_INV5YLL09V1 | NDAR_INVBCHF6V85 | NDAR_INVGM5D6MEW | NDAR_INVN31J6BXZ | NDAR_INVVB32WMEB |
| NDAR_INV0PLKFP06 | NDAR_INV5Z57C4GY | NDAR_INVBCMXWCV0 | NDAR_INVGM7YJH08 | NDAR_INVN394NEWK | NDAR_INVVB6CJP4C |
| NDAR_INV0PR0WY8F | NDAR_INV5ZEMD2C6 | NDAR_INVBD3JGYKK | NDAR_INVGMCEHMN0 | NDAR_INVN3M2X26L | NDAR_INVVB893JHP |
| NDAR_INV0PU19T4K | NDAR_INV5ZKVPJ7T | NDAR_INVBDE6PW3H | NDAR_INVGMFL6ATJ | NDAR_INVN3NK0GNE | NDAR_INVVBHGA5ZP |
| NDAR_INV0R5220TJ | NDAR_INV5ZLLK0YM | NDAR_INVBDHAN68D | NDAR_INVGMJ7KA11 | NDAR_INVN3W1266D | NDAR_INVVBL6F58A |
| NDAR_INV0RA4PBPV | NDAR_INV6012XAJ6 | NDAR_INVBDHFHJUN | NDAR_INVGMRMU1HU | NDAR_INVN4800E3M | NDAR_INVVBM9M2WA |
| NDAR_INV0U23M45G | NDAR_INV60VLVXBP | NDAR_INVBDMDX4EW | NDAR_INVGMVWPXD7 | NDAR_INVN4YXHNL8 | NDAR_INVVC130UV5 |
| NDAR_INV0UA196B6 | NDAR_INV60WMW4VN | NDAR_INVBDMGZK3B | NDAR_INVGN20EAHW | NDAR_INVN52TVLYF | NDAR_INVVC1FVHRL |
| NDAR_INV0UEA3Z9E | NDAR_INV618RC28E | NDAR_INVBDTGBXYZ | NDAR_INVGNBTJYEB | NDAR_INVN53926NR | NDAR_INVVCEAYC27 |
| NDAR_INV0UL9YFZJ | NDAR_INV619DWVEK | NDAR_INVBE6KKTH2 | NDAR_INVGNHB07CM | NDAR_INVN542D50W | NDAR_INVVCWLD1YC |
| NDAR_INV0UWD26VV | NDAR_INV61GCTA64 | NDAR_INVBEADWGRU | NDAR_INVGP88W2WM | NDAR_INVN5BG5LUD | NDAR_INVVDG1UULL |
| NDAR_INV0UYDV4HJ | NDAR_INV61GRLR8M | NDAR_INVBEG60AUC | NDAR_INVGPLJDFMK | NDAR_INVN5H1K388 | NDAR_INVVDT3APC1 |
| NDAR_INV0V1TNU11 | NDAR_INV61K56BR1 | NDAR_INVBEGMET5J | NDAR_INVGPLYJB8P | NDAR_INVN6E9XK0R | NDAR_INVVE6MPVNE |
| NDAR_INV0VXEC29A | NDAR_INV61PF7E1L | NDAR_INVBF0NYFPE | NDAR_INVGPPBRH6H | NDAR_INVN6F1W041 | NDAR_INVVEN6KLB1 |
| NDAR_INV0WYCV590 | NDAR_INV61VV3W6U | NDAR_INVBF8H4PR1 | NDAR_INVGR223RR6 | NDAR_INVN6R4KJPT | NDAR_INVVEVJRL88 |
| NDAR_INV0X02CUCY | NDAR_INV61VX090A | NDAR_INVBFGLJHYJ | NDAR_INVGRKWJ3ET | NDAR_INVN6UYBJ3E | NDAR_INVVEWLJY3Z |
| NDAR_INV0XH07MZ8 | NDAR_INV61X565FX | NDAR_INVBFXU68F9 | NDAR_INVGRZPCR43 | NDAR_INVN72UK9CX | NDAR_INVVF539UBP |
| NDAR_INV0Y9GR364 | NDAR_INV61ZHGXE4 | NDAR_INVBG040P9Z | NDAR_INVGT4WJT38 | NDAR_INVN73BT0HM | NDAR_INVVFC957NV |
| NDAR_INV0YGRZTUF | NDAR_INV620B7YP2 | NDAR_INVBG2Z79LC | NDAR_INVGUWJN86W | NDAR_INVN8KGV116 | NDAR_INVVFCBH31R |
| NDAR_INV0YLLNXRL | NDAR_INV625BWGDB | NDAR_INVBG59D5MV | NDAR_INVGV6LZX0T | NDAR_INVN8VXKZGH | NDAR_INVVFMTWZYL |
| NDAR_INV0YWBYAGP | NDAR_INV62P1BPWE | NDAR_INVBG7FNXZB | NDAR_INVGVAR0AFB | NDAR_INVN98L0THW | NDAR_INVVFX02WGJ |
| NDAR_INV0ZAYELPK | NDAR_INV630AFLN3 | NDAR_INVBG7ZM9D2 | NDAR_INVGVC01P7F | NDAR_INVN98XYXM3 | NDAR_INVVG1MF8K5 |
| NDAR_INV0ZTJ2432 | NDAR_INV63H879ZC | NDAR_INVBGD53R5K | NDAR_INVGVDCETLW | NDAR_INVN9D4XZKE | NDAR_INVVG3X2TFW |
| NDAR_INV107UCJ69 | NDAR_INV63JMZ9HT | NDAR_INVBGYRJERP | NDAR_INVGW6CFXWJ | NDAR_INVNAFXDF3U | NDAR_INVVGBGVFYF |
| NDAR_INV10DN9UHY | NDAR_INV64EVZUTB | NDAR_INVBHA692K1 | NDAR_INVGW77XDKC | NDAR_INVNAJWH1CY | NDAR_INVVGFCTD9T |
| NDAR_INV10KXDDBK | NDAR_INV64PE3J4T | NDAR_INVBHCAN3DK | NDAR_INVGW88GH5L | NDAR_INVNB03VB7J | NDAR_INVVH4YPG9H |
| NDAR_INV10MWD99M | NDAR_INV657UW3RF | NDAR_INVBHHEUCLB | NDAR_INVGWF3GZ8C | NDAR_INVNB6EVP2A | NDAR_INVVHBYV2NC |
| NDAR_INV11G4DZFX | NDAR_INV65NLFHYD | NDAR_INVBHM0HRB4 | NDAR_INVGWGVANWJ | NDAR_INVNBCLFXHV | NDAR_INVVHKDUKTN |
| NDAR_INV11THDW9B | NDAR_INV65P1JE5L | NDAR_INVBJ0A0JN2 | NDAR_INVGWHUZZZC | NDAR_INVNBJ852WE | NDAR_INVVHUVKEG1 |
| NDAR_INV11UF2ABG | NDAR_INV65P8PM1D | NDAR_INVBJVF8AGB | NDAR_INVGX2KR8D2 | NDAR_INVNBJHR1RV | NDAR_INVVJ521M24 |
| NDAR_INV11ZVVFHK | NDAR_INV65V1Z314 | NDAR_INVBJYZ741F | NDAR_INVGX6XFWRU | NDAR_INVNBMXW84N | NDAR_INVVJ7A5WBE |
| NDAR_INV1285PMCK | NDAR_INV65X59CTR | NDAR_INVBK3K7AN8 | NDAR_INVGXB5Z3TF | NDAR_INVNBU3UA23 | NDAR_INVVJELFB43 |
| NDAR_INV12CUM0KV | NDAR_INV666M5VR8 | NDAR_INVBK5APVLC | NDAR_INVGXCZ2ELG | NDAR_INVNC0VMHJK | NDAR_INVVJG59G6B |
| NDAR_INV1306ZGW1 | NDAR_INV66ANGUXW | NDAR_INVBK8KBAZM | NDAR_INVGXJCR6C7 | NDAR_INVNCEE4GWH | NDAR_INVVKCT9VLF |
| NDAR_INV1313F6FM | NDAR_INV66W9RTUH | NDAR_INVBKAL413B | NDAR_INVGXU8LUL5 | NDAR_INVNCEUKV27 | NDAR_INVVKLJZA5A |
| NDAR_INV1378XGLA | NDAR_INV66XU9ULV | NDAR_INVBKNLWG3Z | NDAR_INVGXUMDLPL | NDAR_INVNDCR1NEX | NDAR_INVVKMDG53B |
| NDAR_INV138RG20T | NDAR_INV670CPHWJ | NDAR_INVBKR2UJL2 | NDAR_INVGY9L0FCK | NDAR_INVNDLB4UTU | NDAR_INVVKR3UP6G |
| NDAR_INV13LFMTL6 | NDAR_INV677ED18Z | NDAR_INVBKW33JVR | NDAR_INVGYHXT5DV | NDAR_INVNDTALD7T | NDAR_INVVKV5RYVF |
| NDAR_INV13NMXD4V | NDAR_INV6790DE8Z | NDAR_INVBLD8K10D | NDAR_INVGYYJH5RH | NDAR_INVNDTCY532 | NDAR_INVVL42KJTZ |
| NDAR_INV13PRF0TM | NDAR_INV67JE45TU | NDAR_INVBLJJA3KF | NDAR_INVGZ1H046E | NDAR_INVNEM5D36M | NDAR_INVVLHZP6FR |
| NDAR_INV145EPB4G | NDAR_INV67TUZJU7 | NDAR_INVBLX310KU | NDAR_INVGZJDGF8L | NDAR_INVNERE3UUN | NDAR_INVVLXFA650 |
| NDAR_INV14992W6G | NDAR_INV68DDW0WH | NDAR_INVBLZ7H5LD | NDAR_INVGZMP9AHJ | NDAR_INVNFBP257T | NDAR_INVVLZ1FB8Y |
| NDAR_INV14UJARAJ | NDAR_INV68L0WZRB | NDAR_INVBMFRB748 | NDAR_INVH0474HRG | NDAR_INVNGAXVYPV | NDAR_INVVM0AGJA5 |
| NDAR_INV158CYMLK | NDAR_INV68TA7J93 | NDAR_INVBMMEGVWT | NDAR_INVH04KW20B | NDAR_INVNGKCJE4V | NDAR_INVVMNZP2RA |
| NDAR_INV15M33G49 | NDAR_INV68UE7DYK | NDAR_INVBMMM82AG | NDAR_INVH0DGUW54 | NDAR_INVNGL4MJW6 | NDAR_INVVN646T2K |
| NDAR_INV15MFU6UZ | NDAR_INV69UKEYVY | NDAR_INVBMTA765G | NDAR_INVH1CG52NU | NDAR_INVNGUM0P6A | NDAR_INVVNAHZMFZ |
| NDAR_INV15VX8FJL | NDAR_INV69W6GH2W | NDAR_INVBMTP1HD9 | NDAR_INVH1JMR2EA | NDAR_INVNHZ8AAGB | NDAR_INVVNRTKY5K |
| NDAR_INV16PJ1RJ6 | NDAR_INV6A2D8DTU | NDAR_INVBN1EY1AM | NDAR_INVH1U9GLWT | NDAR_INVNJA6E3NT | NDAR_INVVPBZABW8 |
| NDAR_INV170X8DA0 | NDAR_INV6ABHCVYT | NDAR_INVBNBF1GWB | NDAR_INVH2CGXAAN | NDAR_INVNJGJ5FRB | NDAR_INVVPCKCXWA |
| NDAR_INV174DUV2F | NDAR_INV6AC2J61C | NDAR_INVBNRN2W15 | NDAR_INVH2CX87U1 | NDAR_INVNJJKF0G9 | NDAR_INVVPDNL3D5 |
| NDAR_INV17UGGHGJ | NDAR_INV6ARV4JZP | NDAR_INVBNW6NPRK | NDAR_INVH2D9U5VD | NDAR_INVNJRMP62L | NDAR_INVVPFWLP9R |
| NDAR_INV17UL1T8L | NDAR_INV6BB3VGEP | NDAR_INVBPB9W02P | NDAR_INVH2JCLXCG | NDAR_INVNJZ90LJV | NDAR_INVVPNP73C4 |
| NDAR_INV18KWP8N1 | NDAR_INV6BVVAY29 | NDAR_INVBPPLUGKN | NDAR_INVH30DZ5Z7 | NDAR_INVNKCFGVZJ | NDAR_INVVPY3B7XG |
| NDAR_INV18RUWAJN | NDAR_INV6BWXMYPY | NDAR_INVBR0V5H2X | NDAR_INVH32GHURA | NDAR_INVNKL9ZDUT | NDAR_INVVPZM5DG3 |
| NDAR_INV18XYL4BV | NDAR_INV6CG96F0T | NDAR_INVBR1YZPV4 | NDAR_INVH37LMD3K | NDAR_INVNKNMHEML | NDAR_INVVR3KXTRE |
| NDAR_INV1925AD9X | NDAR_INV6CNL0TLZ | NDAR_INVBRRAE16V | NDAR_INVH3V74X88 | NDAR_INVNKW63Z0P | NDAR_INVVR534N8K |
| NDAR_INV19BR9YHW | NDAR_INV6CUNP3EL | NDAR_INVBT1JM1WA | NDAR_INVH43585GP | NDAR_INVNKWEHXRD | NDAR_INVVR6JW9BP |
| NDAR_INV19JV1ZX5 | NDAR_INV6CZ8A7P2 | NDAR_INVBTA2BMZ6 | NDAR_INVH52P6NHR | NDAR_INVNKYNEYLV | NDAR_INVVR93TB4C |
| NDAR_INV19YE2JCZ | NDAR_INV6D3TTBKJ | NDAR_INVBTABKVCU | NDAR_INVH5344TM1 | NDAR_INVNLE5Z05F | NDAR_INVVRDPAL09 |
| NDAR_INV19ZM2GTU | NDAR_INV6D9PKM7V | NDAR_INVBTLEZJL0 | NDAR_INVH5JWK5CC | NDAR_INVNLL5U5K7 | NDAR_INVVRYNAJ4H |
| NDAR_INV1A3NCEUP | NDAR_INV6DYLLUU6 | NDAR_INVBTTCJX9R | NDAR_INVH5VGWKZU | NDAR_INVNM81RNFR | NDAR_INVVTKJGFA3 |
| NDAR_INV1A8NWWZG | NDAR_INV6E5JG9U4 | NDAR_INVBU2E2YDP | NDAR_INVH679EZ0Z | NDAR_INVNN1MW2UN | NDAR_INVVUP2JXUY |
| NDAR_INV1AFNKM8M | NDAR_INV6ELMHVPX | NDAR_INVBU31791C | NDAR_INVH6E8DVLN | NDAR_INVNNC4ZB88 | NDAR_INVVVAATZ9K |
| NDAR_INV1AJNN0V2 | NDAR_INV6EVBBMKN | NDAR_INVBU9KJZX7 | NDAR_INVH6FMF28J | NDAR_INVNNNG8Z3V | NDAR_INVVVD39H82 |
| NDAR_INV1AYXT588 | NDAR_INV6F2ZB4BH | NDAR_INVBUBD3WNE | NDAR_INVH6MRC067 | NDAR_INVNNNX0496 | NDAR_INVVVUL3N89 |
| NDAR_INV1BHHLZ52 | NDAR_INV6FD78Z13 | NDAR_INVBUEMRDVF | NDAR_INVH6PKTBWM | NDAR_INVNNZDV7GK | NDAR_INVVWUJFEDA |
| NDAR_INV1BR989ED | NDAR_INV6FH1EZP6 | NDAR_INVBUPETT7Z | NDAR_INVH712DY5X | NDAR_INVNP03A4NN | NDAR_INVVWYK9ZMX |
| NDAR_INV1BWHMRFY | NDAR_INV6G091ALC | NDAR_INVBUZ6HVF0 | NDAR_INVH75P81TF | NDAR_INVNP2BVPR0 | NDAR_INVVX7ZG792 |
| NDAR_INV1CKXVV32 | NDAR_INV6G0MHGHA | NDAR_INVBV461JTB | NDAR_INVH7X044A7 | NDAR_INVNP9P3E2H | NDAR_INVVXHYLDG8 |
| NDAR_INV1CRC8UTU | NDAR_INV6G63P8W4 | NDAR_INVBV7R8DVW | NDAR_INVH8CK6W6R | NDAR_INVNPCKJF8R | NDAR_INVVXJBWAPD |
| NDAR_INV1D42VFML | NDAR_INV6G6L9NXY | NDAR_INVBVX28AFV | NDAR_INVH8W4FZ68 | NDAR_INVNPNAGWRF | NDAR_INVVXKX7B5H |
| NDAR_INV1D95ZF1L | NDAR_INV6GLTPT5T | NDAR_INVBVYD3AAF | NDAR_INVH93DJ60C | NDAR_INVNR9XMXRM | NDAR_INVVXR1276X |
| NDAR_INV1E2PBKXL | NDAR_INV6H7448TR | NDAR_INVBW02TMBE | NDAR_INVH93P807E | NDAR_INVNRDK253J | NDAR_INVVY3ZED85 |
| NDAR_INV1E4F8UMB | NDAR_INV6HGWZFVC | NDAR_INVBW17AA1F | NDAR_INVH9BEEBTX | NDAR_INVNRKEL6CB | NDAR_INVVYUKADTG |
| NDAR_INV1ECBLYGG | NDAR_INV6HH7ZULZ | NDAR_INVBW96UH0D | NDAR_INVH9DY5DU5 | NDAR_INVNRMA4NRD | NDAR_INVVZ1LY5EP |
| NDAR_INV1ELP5894 | NDAR_INV6HTXA830 | NDAR_INVBWT96BWH | NDAR_INVH9F934NE | NDAR_INVNRVAZLB0 | NDAR_INVVZ37D2G7 |
| NDAR_INV1ERNH60J | NDAR_INV6HVN64HM | NDAR_INVBX1Z1PJ9 | NDAR_INVH9U0HZ23 | NDAR_INVNRZ3PD4C | NDAR_INVVZJ1V3A9 |
| NDAR_INV1ERVN8M1 | NDAR_INV6HZWUM71 | NDAR_INVBXR1L7N8 | NDAR_INVHA2UFAL2 | NDAR_INVNT6BJVLB | NDAR_INVVZJMVTTC |
| NDAR_INV1ETGGL9R | NDAR_INV6J2LL0DX | NDAR_INVBXY02162 | NDAR_INVHAD1KFV7 | NDAR_INVNT6CA1D1 | NDAR_INVVZM2PPGN |
| NDAR_INV1EZ26N40 | NDAR_INV6J2PR0YF | NDAR_INVBY68RNKJ | NDAR_INVHAL0EPJL | NDAR_INVNTBRC1TA | NDAR_INVVZPWCZ82 |
| NDAR_INV1F953XCP | NDAR_INV6J4KCFWV | NDAR_INVBY8XVNJT | NDAR_INVHB1RV7A1 | NDAR_INVNTY8NW4G | NDAR_INVVZYWE87C |
| NDAR_INV1FGBFDT9 | NDAR_INV6JA3GMT0 | NDAR_INVBY912AK4 | NDAR_INVHBD1ZUWB | NDAR_INVNU26TCT4 | NDAR_INVW01VXPJ7 |
| NDAR_INV1FGX3DL8 | NDAR_INV6JF8WUYT | NDAR_INVBZ7YAT48 | NDAR_INVHBDZY8K2 | NDAR_INVNUMKHR3C | NDAR_INVW0BM9LJ7 |
| NDAR_INV1GDR72XA | NDAR_INV6JFY5LBB | NDAR_INVBZA5F2M2 | NDAR_INVHCCKW1DN | NDAR_INVNUVYJFN0 | NDAR_INVW0VV53Y4 |
| NDAR_INV1GJJVVLZ | NDAR_INV6JJFGCTX | NDAR_INVBZZ8KWTC | NDAR_INVHCE1FFDZ | NDAR_INVNUW6H30L | NDAR_INVW17JBT47 |
| NDAR_INV1GK73EHE | NDAR_INV6K35WWL0 | NDAR_INVC0KCZG24 | NDAR_INVHD01JT5V | NDAR_INVNV27DF2W | NDAR_INVW194FUUP |
| NDAR_INV1GKYPGE4 | NDAR_INV6KCEDZAY | NDAR_INVC0LR31N5 | NDAR_INVHD40HGZ7 | NDAR_INVNVHZ41TU | NDAR_INVW1EJC2L6 |
| NDAR_INV1GTKW6ZG | NDAR_INV6KCMKK84 | NDAR_INVC0M1UAY5 | NDAR_INVHD7WG7PA | NDAR_INVNVVMEGAA | NDAR_INVW1N53GX9 |
| NDAR_INV1GVYL05C | NDAR_INV6KKX9JVK | NDAR_INVC0V8F883 | NDAR_INVHD8GZZ47 | NDAR_INVNVY8KZHN | NDAR_INVW22EGAEE |
| NDAR_INV1GXY5VKK | NDAR_INV6KL1B4H0 | NDAR_INVC19XGK75 | NDAR_INVHD91CMNX | NDAR_INVNVZ6Y707 | NDAR_INVW236WLM5 |
| NDAR_INV1GYJNY83 | NDAR_INV6KP3MKL1 | NDAR_INVC1TLGE8E | NDAR_INVHEABF6E2 | NDAR_INVNW8TFAV3 | NDAR_INVW2DZTWZ2 |
| NDAR_INV1H7NZZZ9 | NDAR_INV6KRBKZYB | NDAR_INVC260Z0HT | NDAR_INVHELBYLF5 | NDAR_INVNWUL0U0R | NDAR_INVW2RV7L97 |
| NDAR_INV1HKCTUW1 | NDAR_INV6KUUNE55 | NDAR_INVC2ZW147D | NDAR_INVHF0XT8CF | NDAR_INVNWYK8ZNE | NDAR_INVW2V4XRVC |
| NDAR_INV1HN9LN7W | NDAR_INV6KW88EPW | NDAR_INVC331WXR7 | NDAR_INVHF7RHL7L | NDAR_INVNX6AEDBV | NDAR_INVW305M668 |
| NDAR_INV1J2LV4NY | NDAR_INV6KZZZ5WU | NDAR_INVC39CENF6 | NDAR_INVHFHTPJ5M | NDAR_INVNX6KVGNJ | NDAR_INVW323P74D |
| NDAR_INV1JGK90HZ | NDAR_INV6L03215H | NDAR_INVC3N3P450 | NDAR_INVHFLEU627 | NDAR_INVNX9NHTTU | NDAR_INVW36EDDBL |
| NDAR_INV1JLAATTP | NDAR_INV6L19M78W | NDAR_INVC3Y8MBZ8 | NDAR_INVHFTLY3CY | NDAR_INVNXBPU3GK | NDAR_INVW39H8UW6 |
| NDAR_INV1JU1CGGB | NDAR_INV6LNG5JWF | NDAR_INVC4056LRF | NDAR_INVHG7XVL8P | NDAR_INVNXE35ZCX | NDAR_INVW3LL7MG1 |
| NDAR_INV1K1285TU | NDAR_INV6M1Y11R0 | NDAR_INVC4EAMHZ7 | NDAR_INVHGNA569Y | NDAR_INVNY4HU8H1 | NDAR_INVW49LW8G9 |
| NDAR_INV1KAEAHY9 | NDAR_INV6MB1G78W | NDAR_INVC4GYKW12 | NDAR_INVHHJXG6Y1 | NDAR_INVNY5R8N51 | NDAR_INVW4NWZJP5 |
| NDAR_INV1L5VJRZG | NDAR_INV6MDP38L0 | NDAR_INVC4HKHURN | NDAR_INVHHP7PPUR | NDAR_INVNY9E48W0 | NDAR_INVW4PDFHV1 |
| NDAR_INV1LC1VRP3 | NDAR_INV6MRZ56RM | NDAR_INVC4KP3KCA | NDAR_INVHJ09MKKG | NDAR_INVNZ2C50P3 | NDAR_INVW4W8HGP2 |
| NDAR_INV1LW4GJ4L | NDAR_INV6N349E4J | NDAR_INVC4M8E621 | NDAR_INVHJ8TZ0PJ | NDAR_INVNZAAFPBG | NDAR_INVW58F28HT |
| NDAR_INV1MHAP6AR | NDAR_INV6NJ4UWH8 | NDAR_INVC4XAJGAN | NDAR_INVHJDFAMYD | NDAR_INVP023WXRZ | NDAR_INVW5LV1C4M |
| NDAR_INV1ML5RVV4 | NDAR_INV6NKPMPRL | NDAR_INVC53FTV59 | NDAR_INVHJEXD2VN | NDAR_INVP0XRDYT0 | NDAR_INVW6GWG9J4 |
| NDAR_INV1MM7DZYV | NDAR_INV6NR4NZWL | NDAR_INVC59Y1E3K | NDAR_INVHJJ00HZ2 | NDAR_INVP12BW7UL | NDAR_INVW6KT5GEB |
| NDAR_INV1MYF7VFL | NDAR_INV6NRB61V1 | NDAR_INVC5B20020 | NDAR_INVHJX7K8DN | NDAR_INVP205AX39 | NDAR_INVW7WRCR3R |
| NDAR_INV1NBATKW5 | NDAR_INV6NUTPPMD | NDAR_INVC5G293GX | NDAR_INVHK13M3T4 | NDAR_INVP2G0PXCM | NDAR_INVW7YX6WG3 |
| NDAR_INV1NBW9RH8 | NDAR_INV6PEZ2Z0K | NDAR_INVC5N9X6VG | NDAR_INVHK68MTVB | NDAR_INVP2M60U74 | NDAR_INVW85HKBUY |
| NDAR_INV1NJK6BVY | NDAR_INV6PUUHUTG | NDAR_INVC63Z86E8 | NDAR_INVHLBDK2HU | NDAR_INVP32CZKYP | NDAR_INVW8HHGURF |
| NDAR_INV1P86DC69 | NDAR_INV6PYRR639 | NDAR_INVC68NXGHP | NDAR_INVHLC0KBEC | NDAR_INVP3UGFYAT | NDAR_INVW8W3GAH9 |
| NDAR_INV1PATDM0H | NDAR_INV6RPBF276 | NDAR_INVC6KRAHVE | NDAR_INVHLLPEWJH | NDAR_INVP3WMBTEH | NDAR_INVW94TKCDX |
| NDAR_INV1PCGZ8YF | NDAR_INV6T7K85JU | NDAR_INVC6ZT38L4 | NDAR_INVHM2TZ8AD | NDAR_INVP3YKZ978 | NDAR_INVW9750ZMA |
| NDAR_INV1PE2WZWB | NDAR_INV6UTCM96K | NDAR_INVC7BXUG5T | NDAR_INVHMM33T0B | NDAR_INVP3Z6VCWP | NDAR_INVWAFKK7PK |
| NDAR_INV1R1AVH6W | NDAR_INV6VZBKHPK | NDAR_INVC7TDBVV1 | NDAR_INVHNJL7TVB | NDAR_INVP45PB0NA | NDAR_INVWAN79D4A |
| NDAR_INV1R4KE1UN | NDAR_INV6W2Z90PJ | NDAR_INVC868NEEC | NDAR_INVHNR4UJ6G | NDAR_INVP4E09BXB | NDAR_INVWAVA878R |
| NDAR_INV1R5PJCK4 | NDAR_INV6WB14N47 | NDAR_INVC88CYTJE | NDAR_INVHNU1D3NL | NDAR_INVP4LTE255 | NDAR_INVWAXXJPLR |
| NDAR_INV1R7RYN1P | NDAR_INV6WJ57CC2 | NDAR_INVC94L2H0F | NDAR_INVHP3KMLEB | NDAR_INVP4X701TR | NDAR_INVWB0280RB |
| NDAR_INV1T61M03G | NDAR_INV6WW1A9ER | NDAR_INVC9DWTY95 | NDAR_INVHP4DCPG2 | NDAR_INVP598P7PM | NDAR_INVWB0RVAGY |
| NDAR_INV1TM2HNZW | NDAR_INV6X19N1LP | NDAR_INVC9N11UXX | NDAR_INVHRNX3BK4 | NDAR_INVP5BUWK7E | NDAR_INVWB7BXZD6 |
| NDAR_INV1TTVDAJ9 | NDAR_INV6X4L1EPK | NDAR_INVC9ZZ3NYD | NDAR_INVHRUJUH1Y | NDAR_INVP5DWFRD0 | NDAR_INVWBAKLX5Y |
| NDAR_INV1U4C4M56 | NDAR_INV6X9U8P56 | NDAR_INVCA6R8P2J | NDAR_INVHRZ2FCEJ | NDAR_INVP5J0UJXG | NDAR_INVWBEMY06N |
| NDAR_INV1V8J43DP | NDAR_INV6XNN68K1 | NDAR_INVCAL4W4CW | NDAR_INVHT6KEU1H | NDAR_INVP5PE9ZJW | NDAR_INVWBGB46VZ |
| NDAR_INV1V9TCMZW | NDAR_INV6XT4AD09 | NDAR_INVCB9AY93V | NDAR_INVHT9V9KV9 | NDAR_INVP6166U2A | NDAR_INVWBPRXX2G |
| NDAR_INV1VCF7KC6 | NDAR_INV6XTE4VEF | NDAR_INVCBPYR2EY | NDAR_INVHTNVLRBR | NDAR_INVP61N70TM | NDAR_INVWBVTFEHK |
| NDAR_INV1VNU5KTM | NDAR_INV6Y2ZGZRC | NDAR_INVCBV85UMM | NDAR_INVHTWDYYK1 | NDAR_INVP65TV30G | NDAR_INVWC7XWTUD |
| NDAR_INV1VRGHTD2 | NDAR_INV6Y7D78GZ | NDAR_INVCCBAJKLY | NDAR_INVHU28FNBP | NDAR_INVP69PZ8N4 | NDAR_INVWCLDPKM7 |
| NDAR_INV1VVMK6CP | NDAR_INV6Y7LCCWT | NDAR_INVCCBRA37Z | NDAR_INVHUD7MU7R | NDAR_INVP6EV2WUA | NDAR_INVWDCENDZ9 |
| NDAR_INV1VXVRPHJ | NDAR_INV6YFHHNGB | NDAR_INVCD63NC88 | NDAR_INVHUWXZLZA | NDAR_INVP6NAG8DY | NDAR_INVWDLCWZL7 |
| NDAR_INV1WED9TPM | NDAR_INV6YMY7HCD | NDAR_INVCDNXAUT3 | NDAR_INVHV5ACGVN | NDAR_INVP79AXMBD | NDAR_INVWDV4WJ1V |
| NDAR_INV1WG8AL3V | NDAR_INV6Z1RJLBY | NDAR_INVCE0TWGXM | NDAR_INVHV9VDD6H | NDAR_INVP7NCC1J0 | NDAR_INVWE1DE80Z |
| NDAR_INV1WJX687U | NDAR_INV7012EA0L | NDAR_INVCE16DXG2 | NDAR_INVHVAJVTNH | NDAR_INVP7UFXE5A | NDAR_INVWE5GN0XG |
| NDAR_INV1WVHVGH7 | NDAR_INV701F04JM | NDAR_INVCE9MY4X4 | NDAR_INVHVLKT4A9 | NDAR_INVP7ZDJ6UZ | NDAR_INVWE96D8HC |
| NDAR_INV1X8K8NC3 | NDAR_INV702TNTVC | NDAR_INVCEWUA63Z | NDAR_INVHW0PPV8W | NDAR_INVP8UTHGXY | NDAR_INVWE9H9FRJ |
| NDAR_INV1XAZP6R9 | NDAR_INV7063065M | NDAR_INVCFCXNV7G | NDAR_INVHWPUPM4D | NDAR_INVP8WE2JYW | NDAR_INVWEJMEVJN |
| NDAR_INV1XGKZHVG | NDAR_INV70EJ1089 | NDAR_INVCFK3HDCF | NDAR_INVHWTVMKM0 | NDAR_INVP8Z5RP3Y | NDAR_INVWELLZDW7 |
| NDAR_INV1XH2YX3W | NDAR_INV71KVYY5T | NDAR_INVCFT8L45Y | NDAR_INVHX1RU4HV | NDAR_INVP93WF83K | NDAR_INVWEV9M1BE |
| NDAR_INV1Y1Z29D0 | NDAR_INV72Z70LY1 | NDAR_INVCFTDDBW7 | NDAR_INVHX5PF696 | NDAR_INVP9L68A4A | NDAR_INVWFF7HRUN |
| NDAR_INV1Y2L6GWB | NDAR_INV735T3VEK | NDAR_INVCG1XBZFD | NDAR_INVHXFHEYX7 | NDAR_INVP9LDNE6V | NDAR_INVWFN7K4C5 |
| NDAR_INV1Y3BE09E | NDAR_INV73C9ZTFF | NDAR_INVCG7XE3KT | NDAR_INVHXH48HLR | NDAR_INVP9NTMNTN | NDAR_INVWFP0E099 |
| NDAR_INV1YE699WK | NDAR_INV73P1LVTY | NDAR_INVCG8XPP5D | NDAR_INVHXL07E2D | NDAR_INVPA1L60YE | NDAR_INVWFTWLBY6 |
| NDAR_INV1YFEDW44 | NDAR_INV743YNW7Y | NDAR_INVCGHNP1DK | NDAR_INVHXUU4ET3 | NDAR_INVPA8MT8ZF | NDAR_INVWG4ZLXZY |
| NDAR_INV1YGWGEYE | NDAR_INV7466RMUA | NDAR_INVCGXC468Y | NDAR_INVHY5L5CMP | NDAR_INVPATP4RPG | NDAR_INVWGYVBK6Y |
| NDAR_INV1YH0352V | NDAR_INV749XW1TD | NDAR_INVCJ001M61 | NDAR_INVHZ0C17PZ | NDAR_INVPAXB8Z2R | NDAR_INVWGZNJCT7 |
| NDAR_INV1ZD6AF6H | NDAR_INV74A66FHC | NDAR_INVCJ5UYUBX | NDAR_INVHZ7D1EYU | NDAR_INVPAXW8WZB | NDAR_INVWH0P4JHC |
| NDAR_INV1ZMK9GCG | NDAR_INV74MXUMZP | NDAR_INVCJ8FE0MN | NDAR_INVHZDNK5V8 | NDAR_INVPBG592EC | NDAR_INVWH7B6J1P |
| NDAR_INV1ZPJCKVC | NDAR_INV75APGWF2 | NDAR_INVCJHJMM1A | NDAR_INVHZFMKD7L | NDAR_INVPBVUXEJZ | NDAR_INVWH7LABN8 |
| NDAR_INV20CZ5PTX | NDAR_INV75H3LM5P | NDAR_INVCJW0RAFP | NDAR_INVHZFXERBU | NDAR_INVPBYWZ1LX | NDAR_INVWJ086JLF |
| NDAR_INV20GDJPEY | NDAR_INV75Y0PRA7 | NDAR_INVCK8BV0JF | NDAR_INVHZNNE6X0 | NDAR_INVPCFM6DWK | NDAR_INVWJ1LWK8J |
| NDAR_INV20MDTUZ8 | NDAR_INV75ZYAV1X | NDAR_INVCKA8AFM8 | NDAR_INVHZR13VNX | NDAR_INVPCHJT5DP | NDAR_INVWJ2YVYHW |
| NDAR_INV20NDWAH8 | NDAR_INV765GH2MK | NDAR_INVCKDH5018 | NDAR_INVHZUEB7Z2 | NDAR_INVPCJWLN13 | NDAR_INVWJ9EAVVK |
| NDAR_INV21BW63DB | NDAR_INV7692F35W | NDAR_INVCL748RXE | NDAR_INVJ08J6XCZ | NDAR_INVPCLAGP3Z | NDAR_INVWJDKPLBG |
| NDAR_INV21CN9606 | NDAR_INV76K5LEL6 | NDAR_INVCLPUR48B | NDAR_INVJ0Y53RRE | NDAR_INVPD2HB0LU | NDAR_INVWK84XHKV |
| NDAR_INV21VF36AV | NDAR_INV76NR9YPR | NDAR_INVCMGFWG2E | NDAR_INVJ19WCNEJ | NDAR_INVPDDWCGGW | NDAR_INVWKGFMVUC |
| NDAR_INV22GE0DMV | NDAR_INV76XDC3CZ | NDAR_INVCMPRKMKC | NDAR_INVJ1XKYGP9 | NDAR_INVPDFDEURE | NDAR_INVWL2BVWEJ |
| NDAR_INV22LW15TV | NDAR_INV77M916PU | NDAR_INVCN7V5XKE | NDAR_INVJ1Y3Z20G | NDAR_INVPDHM8VFC | NDAR_INVWL6W9JWZ |
| NDAR_INV22VV8H44 | NDAR_INV788NL0CN | NDAR_INVCN8DWC2V | NDAR_INVJ27L7Z6G | NDAR_INVPDTCVTVC | NDAR_INVWLJKU8MX |
| NDAR_INV230CTHZE | NDAR_INV78L0ZLZW | NDAR_INVCNCRDPTT | NDAR_INVJ2CBCRYX | NDAR_INVPDUZETD1 | NDAR_INVWLNTC8GU |
| NDAR_INV233UX0N5 | NDAR_INV79NF4B2M | NDAR_INVCP16BG12 | NDAR_INVJ37ZWA6X | NDAR_INVPDVYFN7E | NDAR_INVWLX2MF41 |
| NDAR_INV2358EMKW | NDAR_INV79ZF8916 | NDAR_INVCP3Z2564 | NDAR_INVJ3DFH4DA | NDAR_INVPE18AEMN | NDAR_INVWM4B6L05 |
| NDAR_INV237NFLEB | NDAR_INV7A80G68A | NDAR_INVCP9MXC42 | NDAR_INVJ3PNH5ZW | NDAR_INVPE8Y8D94 | NDAR_INVWMETUT1U |
| NDAR_INV23XHKMH5 | NDAR_INV7ALJZGKH | NDAR_INVCPJCZKP2 | NDAR_INVJ45PTP2H | NDAR_INVPENZ13BH | NDAR_INVWMM5T2PL |
| NDAR_INV23XZD51D | NDAR_INV7ATXXJUG | NDAR_INVCPT9ZT4B | NDAR_INVJ45XGZRF | NDAR_INVPETLL0NE | NDAR_INVWMPDRV7H |
| NDAR_INV24534L6F | NDAR_INV7B2V2JHN | NDAR_INVCPUDHWZM | NDAR_INVJ4U9BRT9 | NDAR_INVPF3UJTKW | NDAR_INVWMTTWY8K |
| NDAR_INV249JM0NY | NDAR_INV7B9VZ5UU | NDAR_INVCPYLG68G | NDAR_INVJ4YPY232 | NDAR_INVPF5T4C8D | NDAR_INVWMYX7H7U |
| NDAR_INV24BT0Y26 | NDAR_INV7BB38F3D | NDAR_INVCR43W7MR | NDAR_INVJ51UCKWX | NDAR_INVPFB7Y8AM | NDAR_INVWN8UC0FU |
| NDAR_INV24LWV4C5 | NDAR_INV7BJ6UKUT | NDAR_INVCR7WRBTX | NDAR_INVJ5JJZDPB | NDAR_INVPFC26GTC | NDAR_INVWNBZJPCU |
| NDAR_INV24YLW97V | NDAR_INV7BKAZZAM | NDAR_INVCR8X09T2 | NDAR_INVJ5VHD38X | NDAR_INVPFNN111R | NDAR_INVWNTT8WKJ |
| NDAR_INV25PJA9DK | NDAR_INV7BRAYU73 | NDAR_INVCRM6V6NX | NDAR_INVJ5VHG1C3 | NDAR_INVPG04NJDC | NDAR_INVWP0ZYHN3 |
| NDAR_INV25UXU689 | NDAR_INV7BTZH7L2 | NDAR_INVCRY0YLTV | NDAR_INVJ5XPPK6N | NDAR_INVPG5HVR50 | NDAR_INVWP2U8M5Y |
| NDAR_INV263A3T6P | NDAR_INV7C9DD99Y | NDAR_INVCT1P8L38 | NDAR_INVJ5ZCLVEA | NDAR_INVPGRJ289W | NDAR_INVWPL8UB72 |
| NDAR_INV263W8YMY | NDAR_INV7CBUV9AY | NDAR_INVCT3X58XN | NDAR_INVJ652C1TF | NDAR_INVPGW66JN0 | NDAR_INVWPVF0L7H |
| NDAR_INV26E5WLZL | NDAR_INV7CC21C8B | NDAR_INVCT4DY62X | NDAR_INVJ69E194R | NDAR_INVPH3H7TPD | NDAR_INVWR1VYFBV |
| NDAR_INV26MRFWHN | NDAR_INV7D8F79P8 | NDAR_INVCT6N4C6Y | NDAR_INVJ6K2LWCV | NDAR_INVPH4BZ3KW | NDAR_INVWRCZDDAY |
| NDAR_INV26UB5CFA | NDAR_INV7DG2NPET | NDAR_INVCTFL718M | NDAR_INVJ716H2Y0 | NDAR_INVPHGUL1Z3 | NDAR_INVWRETMHUC |
| NDAR_INV279N3WC9 | NDAR_INV7DU2R3J4 | NDAR_INVCU05ND45 | NDAR_INVJ72AUZZW | NDAR_INVPHHNBM9Z | NDAR_INVWRFN7EM0 |
| NDAR_INV27CF9R4C | NDAR_INV7DY4B12E | NDAR_INVCU8K037T | NDAR_INVJ7V2VTL2 | NDAR_INVPJ39YVH9 | NDAR_INVWRLG7NX5 |
| NDAR_INV27GFX2N7 | NDAR_INV7E2LV7HF | NDAR_INVCUABM4D8 | NDAR_INVJ87UYTGU | NDAR_INVPJ7TLN56 | NDAR_INVWRWDR7HE |
| NDAR_INV27H5R87Z | NDAR_INV7E36P23G | NDAR_INVCUGYRU5T | NDAR_INVJ8A4A9P2 | NDAR_INVPJAYKP36 | NDAR_INVWRX5TRT3 |
| NDAR_INV27P8BVEW | NDAR_INV7E8WJTYE | NDAR_INVCUHZ4YBK | NDAR_INVJ8JU5JAA | NDAR_INVPJF841VD | NDAR_INVWT13MP64 |
| NDAR_INV28B537H0 | NDAR_INV7EZLVXDN | NDAR_INVCUPEY1Z0 | NDAR_INVJ9YKZEB3 | NDAR_INVPJP1641H | NDAR_INVWTYHZVG8 |
| NDAR_INV28DR91DW | NDAR_INV7F555HX9 | NDAR_INVCURU4G26 | NDAR_INVJAEV7W5J | NDAR_INVPJWETA8W | NDAR_INVWU3CFG57 |
| NDAR_INV28NRLWRX | NDAR_INV7F8T9HG0 | NDAR_INVCV0BAW89 | NDAR_INVJAUU039W | NDAR_INVPK1G4FMB | NDAR_INVWU4PVYX1 |
| NDAR_INV28X60X09 | NDAR_INV7FG8NTPP | NDAR_INVCV6DK10M | NDAR_INVJB8CRWMV | NDAR_INVPKGZ9380 | NDAR_INVWUHH5YTP |
| NDAR_INV29299DLJ | NDAR_INV7FGBBTWL | NDAR_INVCVAXRBP7 | NDAR_INVJC4Y18YR | NDAR_INVPKHAU8GN | NDAR_INVWUYB82C8 |
| NDAR_INV29RMU5LN | NDAR_INV7GC6PLZG | NDAR_INVCVEWVJ5J | NDAR_INVJC52GNNU | NDAR_INVPKXLA8CU | NDAR_INVWVA9UY95 |
| NDAR_INV29TAPT8B | NDAR_INV7GCR03MF | NDAR_INVCWC14R4P | NDAR_INVJC5NPM3Y | NDAR_INVPL13NA8X | NDAR_INVWVE3Y12H |
| NDAR_INV29Y8ZRVC | NDAR_INV7GMJLBGM | NDAR_INVCWC53CBP | NDAR_INVJC8UD8CP | NDAR_INVPL7VWK07 | NDAR_INVWVM2TW33 |
| NDAR_INV2AKULJ38 | NDAR_INV7GTT6FAU | NDAR_INVCWN52N9L | NDAR_INVJCUCGDCF | NDAR_INVPM21LPZT | NDAR_INVWVWUUCX4 |
| NDAR_INV2AKVW3V7 | NDAR_INV7J8ECUH7 | NDAR_INVCX4YJGUW | NDAR_INVJD6CRBP8 | NDAR_INVPM59P2MU | NDAR_INVWVWWBTF1 |
| NDAR_INV2B7P1TMR | NDAR_INV7J9K5U1L | NDAR_INVCX9VNMT6 | NDAR_INVJDVAX1CU | NDAR_INVPMV3Y0GW | NDAR_INVWWMFVX5N |
| NDAR_INV2BKVEFL8 | NDAR_INV7JERJG3Z | NDAR_INVCXKTLU8R | NDAR_INVJDXKXF8T | NDAR_INVPMYAAKXU | NDAR_INVWWRC2ZP0 |
| NDAR_INV2BUTEB8U | NDAR_INV7JGE0MH9 | NDAR_INVCXN95RV7 | NDAR_INVJDZ7L3H6 | NDAR_INVPN0FY9ER | NDAR_INVWX21FUMG |
| NDAR_INV2C2HN3CL | NDAR_INV7K0DFRE1 | NDAR_INVCXW3Y64K | NDAR_INVJDZ90ATC | NDAR_INVPNF8BAGH | NDAR_INVWX3C6A3L |
| NDAR_INV2CB23NEG | NDAR_INV7L9EAFEX | NDAR_INVCXXT5U2P | NDAR_INVJE33Y96D | NDAR_INVPNUJ3TUX | NDAR_INVWX7WTJX6 |
| NDAR_INV2CL5C6NJ | NDAR_INV7LBEJR41 | NDAR_INVCY1CFU0B | NDAR_INVJE5T60YC | NDAR_INVPNUMLXXA | NDAR_INVWXDY4HKM |
| NDAR_INV2CTVWFH6 | NDAR_INV7LE7FH8R | NDAR_INVCY51KY08 | NDAR_INVJE7U8GZ4 | NDAR_INVPNVM0V32 | NDAR_INVWXGW2FKF |
| NDAR_INV2CVWTNF7 | NDAR_INV7LMEMN09 | NDAR_INVCYD11BRB | NDAR_INVJE8U3WEP | NDAR_INVPPL151XE | NDAR_INVWXLHTR31 |
| NDAR_INV2D6AVBFY | NDAR_INV7LWNRC3F | NDAR_INVCYF28BRB | NDAR_INVJEEWKCB5 | NDAR_INVPPN58EEF | NDAR_INVWXW22AJX |
| NDAR_INV2DHY1CHU | NDAR_INV7MB6N322 | NDAR_INVCZ2UNC0K | NDAR_INVJEFU1LRB | NDAR_INVPPTGWLKJ | NDAR_INVWY6VZCVE |
| NDAR_INV2DLW1KXL | NDAR_INV7MCB12JE | NDAR_INVCZ5MFAJL | NDAR_INVJEUB6WZP | NDAR_INVPPY8DZG0 | NDAR_INVWZ9XXBWY |
| NDAR_INV2DTHE81G | NDAR_INV7MFDRPRV | NDAR_INVCZ89L9CB | NDAR_INVJF3KUA66 | NDAR_INVPPYH7MGJ | NDAR_INVX09F55B8 |
| NDAR_INV2DYU5KZK | NDAR_INV7MZN86PD | NDAR_INVCZWTTCVK | NDAR_INVJF85FC7L | NDAR_INVPR7K4MAW | NDAR_INVX0D8H6J7 |
| NDAR_INV2DZUL8LC | NDAR_INV7N9P7K46 | NDAR_INVD0ETUKJA | NDAR_INVJF8W3PW6 | NDAR_INVPRFZLX5F | NDAR_INVX0KUKVL4 |
| NDAR_INV2EN91V04 | NDAR_INV7NAGRWL5 | NDAR_INVD0TZD25R | NDAR_INVJFJL5WJL | NDAR_INVPRLTKVRN | NDAR_INVX0YRUY7C |
| NDAR_INV2EW24V3A | NDAR_INV7NNTJYGL | NDAR_INVD1C3Z8TZ | NDAR_INVJG8HUWJZ | NDAR_INVPT3LUUN3 | NDAR_INVX1YX7PDR |
| NDAR_INV2F51HZAP | NDAR_INV7P2D519Z | NDAR_INVD1EMLDV6 | NDAR_INVJGFBY8J1 | NDAR_INVPTL35GHE | NDAR_INVX225RA0Y |
| NDAR_INV2F729N9A | NDAR_INV7P8CG4M6 | NDAR_INVD25VAT32 | NDAR_INVJGPTJZAC | NDAR_INVPU92TRNH | NDAR_INVX24GH2TJ |
| NDAR_INV2FL9EYH8 | NDAR_INV7PMXEN0M | NDAR_INVD2C4V9ZT | NDAR_INVJH7UFKKJ | NDAR_INVPUEXNZJU | NDAR_INVX2BLRMU7 |
| NDAR_INV2FMXCB35 | NDAR_INV7PR9WHRN | NDAR_INVD31ULK08 | NDAR_INVJHJDGEFN | NDAR_INVPUKB7WJ3 | NDAR_INVX2MAVLUN |
| NDAR_INV2G3T7XUL | NDAR_INV7PU5X7GA | NDAR_INVD435BDPH | NDAR_INVJJ2DNAR0 | NDAR_INVPUVCADU0 | NDAR_INVX2MNECZ2 |
| NDAR_INV2GRXYP02 | NDAR_INV7R0TF1TJ | NDAR_INVD4J6E7PK | NDAR_INVJJ9NBNC9 | NDAR_INVPV7EDNMA | NDAR_INVX2T12EWL |
| NDAR_INV2H0H09CW | NDAR_INV7R67NVBB | NDAR_INVD4TVEL8N | NDAR_INVJJMH6ZDT | NDAR_INVPV9AKNCF | NDAR_INVX329JYUJ |
| NDAR_INV2H2KZ67D | NDAR_INV7RH75L3L | NDAR_INVD4YYXM1K | NDAR_INVJJWR8W0W | NDAR_INVPVMY2MPT | NDAR_INVX3PYJZPC |
| NDAR_INV2H2UTYYP | NDAR_INV7RPBV53C | NDAR_INVD52N8T9J | NDAR_INVJK6VB601 | NDAR_INVPVT0X3NH | NDAR_INVX3ZCBLYM |
| NDAR_INV2H5KK1DY | NDAR_INV7TENJYLH | NDAR_INVD59G5M7D | NDAR_INVJKEK2N1R | NDAR_INVPWCF8X4C | NDAR_INVX3ZLE0DN |
| NDAR_INV2HYAENE6 | NDAR_INV7TJ9713F | NDAR_INVD5B0X85Y | NDAR_INVJKJXL96V | NDAR_INVPWDDAD3B | NDAR_INVX43TTKW0 |
| NDAR_INV2J50AKJP | NDAR_INV7TJVNX42 | NDAR_INVD5DMDCCC | NDAR_INVJKLMWC89 | NDAR_INVPWDTCU07 | NDAR_INVX4GZFHDA |
| NDAR_INV2JPG2BTW | NDAR_INV7TUNMGNZ | NDAR_INVD5FWJDCY | NDAR_INVJL71L4YC | NDAR_INVPWRY2NMT | NDAR_INVX5326K7A |
| NDAR_INV2JRRX50R | NDAR_INV7TZHEHB0 | NDAR_INVD5U7YYE5 | NDAR_INVJL7XXYD5 | NDAR_INVPX0NC4FE | NDAR_INVX5GGE1B3 |
| NDAR_INV2K16Y0LC | NDAR_INV7U9JKJT0 | NDAR_INVD5UV2RGA | NDAR_INVJLFZWEL3 | NDAR_INVPX3YX562 | NDAR_INVX5L1CX2N |
| NDAR_INV2K3JH38W | NDAR_INV7UTXNP58 | NDAR_INVD5ZMXFG6 | NDAR_INVJM536KCC | NDAR_INVPX4NDUNA | NDAR_INVX64TGR65 |
| NDAR_INV2KBLDWXW | NDAR_INV7UWTN03J | NDAR_INVD681TFUL | NDAR_INVJMLDL425 | NDAR_INVPXF7PDPX | NDAR_INVX6EPR061 |
| NDAR_INV2KJNX54B | NDAR_INV7WETNA45 | NDAR_INVD6AR573N | NDAR_INVJN2G8BWG | NDAR_INVPXNFBFY9 | NDAR_INVX6FUJZVN |
| NDAR_INV2L17MHHT | NDAR_INV7WH96F72 | NDAR_INVD6E70E57 | NDAR_INVJN9LRK2A | NDAR_INVPXPY8J3A | NDAR_INVX6MBE8T4 |
| NDAR_INV2L2B6VXP | NDAR_INV7WM1CF95 | NDAR_INVD6U7VKP6 | NDAR_INVJNWHJTVZ | NDAR_INVPXUVDWE2 | NDAR_INVX6TJJ447 |
| NDAR_INV2L3VG13G | NDAR_INV7X4CGXMP | NDAR_INVD6X8GC05 | NDAR_INVJP9XWZFR | NDAR_INVPXYXMXTH | NDAR_INVX6WEX0YJ |
| NDAR_INV2L40420R | NDAR_INV7X576WFP | NDAR_INVD7969NZ4 | NDAR_INVJPDBRMPL | NDAR_INVPYJ78DEY | NDAR_INVX711YAM8 |
| NDAR_INV2LTU8ZA2 | NDAR_INV7XMDVMD9 | NDAR_INVD7D90WV5 | NDAR_INVJPKAU189 | NDAR_INVPYMGFXEW | NDAR_INVX7GTH1Y9 |
| NDAR_INV2NV4C2CL | NDAR_INV7XRE8WCY | NDAR_INVD7MKAFA0 | NDAR_INVJPMRY00B | NDAR_INVPYX4FBVY | NDAR_INVX7Y693LX |
| NDAR_INV2NYH1JCE | NDAR_INV7XUV8V0N | NDAR_INVD7RR0C8L | NDAR_INVJPV235G4 | NDAR_INVPZ7LHWF8 | NDAR_INVX86DXRAK |
| NDAR_INV2P0HPD40 | NDAR_INV7Y39VTC8 | NDAR_INVD7UZ5ABH | NDAR_INVJPX8NVUZ | NDAR_INVPZ879XG6 | NDAR_INVX8CRJYVP |
| NDAR_INV2P48F0JJ | NDAR_INV7YA52BU1 | NDAR_INVD7XD417L | NDAR_INVJR0F0ERZ | NDAR_INVPZE8ABE3 | NDAR_INVX8PGW7RC |
| NDAR_INV2RCE88HL | NDAR_INV7YCFCH49 | NDAR_INVD7YYK7CY | NDAR_INVJRFFW8YT | NDAR_INVPZHLGHDN | NDAR_INVXB686CP7 |
| NDAR_INV2RLDDFYE | NDAR_INV7YFTLJBG | NDAR_INVD806H93D | NDAR_INVJRK7YJE3 | NDAR_INVPZK0NW7K | NDAR_INVXBFJB18T |
| NDAR_INV2RYEWWRN | NDAR_INV7YM285FW | NDAR_INVD83LZP78 | NDAR_INVJRXPZU5X | NDAR_INVR06UP6NN | NDAR_INVXBHMF9C2 |
| NDAR_INV2T5K2NDJ | NDAR_INV7YU2MX4Y | NDAR_INVD8N6RPYP | NDAR_INVJT28AYHV | NDAR_INVR09UG23J | NDAR_INVXBNU804E |
| NDAR_INV2T6WFDFJ | NDAR_INV7Z7XV0PB | NDAR_INVD8NF2X35 | NDAR_INVJTA9PW0F | NDAR_INVR0AW8RHV | NDAR_INVXCD5ZXAR |
| NDAR_INV2TBMGWU5 | NDAR_INV7ZTZMB99 | NDAR_INVD9BC5CUY | NDAR_INVJTM54RD3 | NDAR_INVR0J4APPX | NDAR_INVXCHYF80L |
| NDAR_INV2TTD2LB2 | NDAR_INV7ZVJYLM3 | NDAR_INVD9KW5TPY | NDAR_INVJU4UKFJC | NDAR_INVR0TYK5V9 | NDAR_INVXD6CMU32 |
| NDAR_INV2U3DNEET | NDAR_INV804KTLAN | NDAR_INVD9L81NY5 | NDAR_INVJUK1TMPK | NDAR_INVR12GH8DH | NDAR_INVXDAPNHPM |
| NDAR_INV2U3P6G9H | NDAR_INV80CBC0L9 | NDAR_INVD9LT4A11 | NDAR_INVJUKBPY9X | NDAR_INVR1927JG7 | NDAR_INVXDNVH6WF |
| NDAR_INV2U59DP6L | NDAR_INV80VX1MHD | NDAR_INVDAF6VRMG | NDAR_INVJV8V3BD5 | NDAR_INVR1KAV63D | NDAR_INVXE1AD04X |
| NDAR_INV2UHTAC1R | NDAR_INV80Z1RNR4 | NDAR_INVDAH3KGYG | NDAR_INVJVAW0ZW5 | NDAR_INVR1TZTUN2 | NDAR_INVXECRNL56 |
| NDAR_INV2UL6H828 | NDAR_INV8179BTRR | NDAR_INVDAL9UY1P | NDAR_INVJVFW1735 | NDAR_INVR22TV84L | NDAR_INVXEHCCY9N |
| NDAR_INV2UWL3APY | NDAR_INV818GME72 | NDAR_INVDAV11B6Z | NDAR_INVJVK809ML | NDAR_INVR238YY1G | NDAR_INVXEKGFN5Y |
| NDAR_INV2V43CKWH | NDAR_INV81M94TWZ | NDAR_INVDAWZ7L6D | NDAR_INVJW8V5WX6 | NDAR_INVR2EC067F | NDAR_INVXER5A63C |
| NDAR_INV2VD87TK5 | NDAR_INV81R15GP2 | NDAR_INVDAYP39E1 | NDAR_INVJWAPD83W | NDAR_INVR2JZ1RD8 | NDAR_INVXEYPED0K |
| NDAR_INV2VGE4HK8 | NDAR_INV82D8H4A0 | NDAR_INVDB241KG5 | NDAR_INVJWCD55M7 | NDAR_INVR2R52JP7 | NDAR_INVXF3EU2H1 |
| NDAR_INV2VGT219A | NDAR_INV82W5UE50 | NDAR_INVDBCH13DE | NDAR_INVJWVF7NPJ | NDAR_INVR3G28E6R | NDAR_INVXFD605G6 |
| NDAR_INV2VJF8M3C | NDAR_INV831V8XME | NDAR_INVDBF7Z5CL | NDAR_INVJXA05086 | NDAR_INVR3VTVCE5 | NDAR_INVXFURZ24F |
| NDAR_INV2VY7YYNW | NDAR_INV83MAXVLU | NDAR_INVDBHYPZ31 | NDAR_INVJXF3WTN6 | NDAR_INVR44LUUEZ | NDAR_INVXGK0BB15 |
| NDAR_INV2W4HNGV7 | NDAR_INV83P4PG3J | NDAR_INVDBKJ96UF | NDAR_INVJXMWYPZB | NDAR_INVR4EUGHE9 | NDAR_INVXH4JV9XL |
| NDAR_INV2W8WT4Y2 | NDAR_INV84AEJJN9 | NDAR_INVDCFU6FKR | NDAR_INVJXNCXJ3W | NDAR_INVR4Z2P93R | NDAR_INVXH5MJUMZ |
| NDAR_INV2X7YEHPB | NDAR_INV84ERWFDR | NDAR_INVDCMG4GTD | NDAR_INVJXT80FVU | NDAR_INVR51XRC0H | NDAR_INVXH99V5FZ |
| NDAR_INV2X8P07FC | NDAR_INV84XXL5T1 | NDAR_INVDCRLF3FV | NDAR_INVJXUKE3JB | NDAR_INVR5VKKRM9 | NDAR_INVXHDAVM50 |
| NDAR_INV2XAHZZ2A | NDAR_INV8506NUKE | NDAR_INVDCRUCJGE | NDAR_INVJYAGWBLV | NDAR_INVR62PJ1EJ | NDAR_INVXHM7W0B1 |
| NDAR_INV2XU3RAMJ | NDAR_INV854Y6WML | NDAR_INVDDKXYJ2D | NDAR_INVJYBB2VH0 | NDAR_INVR6ET5C77 | NDAR_INVXHU70DR0 |
| NDAR_INV2Y5DW30Z | NDAR_INV857VP9C8 | NDAR_INVDE8G5XB3 | NDAR_INVJYUGN1PH | NDAR_INVR6G17MWX | NDAR_INVXJ2454AG |
| NDAR_INV2YBXVCKR | NDAR_INV86B2YCA7 | NDAR_INVDEHN3PH7 | NDAR_INVJZJBNBTC | NDAR_INVR6KT9419 | NDAR_INVXJ31U6MU |
| NDAR_INV2Z2HJFG1 | NDAR_INV86NEYD0L | NDAR_INVDEN6XZLU | NDAR_INVJZJK5N4L | NDAR_INVR6PJMZ5P | NDAR_INVXJ93E5N3 |
| NDAR_INV2Z67G2F3 | NDAR_INV86XLFCDB | NDAR_INVDEPBAJ6L | NDAR_INVJZRV5ANP | NDAR_INVR6PPY0VL | NDAR_INVXJVE0AXP |
| NDAR_INV2ZA2LC3N | NDAR_INV874HFG94 | NDAR_INVDF4CFND3 | NDAR_INVJZUHG8ZL | NDAR_INVR6TLLPMG | NDAR_INVXK4L71C3 |
| NDAR_INV2ZGK3ZBW | NDAR_INV87LECBH8 | NDAR_INVDF9702P2 | NDAR_INVK0RLYCC7 | NDAR_INVR6V7FU8U | NDAR_INVXK6GKFTM |
| NDAR_INV2ZJXDTEL | NDAR_INV87WRYJ7F | NDAR_INVDFB4NL0N | NDAR_INVK0RXG494 | NDAR_INVR7WBBFTB | NDAR_INVXK6KTKFE |
| NDAR_INV302FDTJF | NDAR_INV880EZ4YA | NDAR_INVDFNR4UMM | NDAR_INVK10RYUC1 | NDAR_INVR8LMF308 | NDAR_INVXKA1ABU7 |
| NDAR_INV306EC6TP | NDAR_INV8865H9WX | NDAR_INVDGP7217P | NDAR_INVK15CAZPG | NDAR_INVR9JZP8TC | NDAR_INVXKJEN0E0 |
| NDAR_INV31JTFZ92 | NDAR_INV88PFKACD | NDAR_INVDGTRNE7V | NDAR_INVK1DE6JAR | NDAR_INVR9LMGEVE | NDAR_INVXKYUY7PR |
| NDAR_INV324JE1UG | NDAR_INV88PHXJ3C | NDAR_INVDGVADKJA | NDAR_INVK1GY1F0R | NDAR_INVR9MG0HJ4 | NDAR_INVXL4UCXMM |
| NDAR_INV32B70T4V | NDAR_INV88RGRPEF | NDAR_INVDGW9RHNK | NDAR_INVK1KZZ8D4 | NDAR_INVRAC0NR51 | NDAR_INVXLF39KRG |
| NDAR_INV32P8UGE7 | NDAR_INV89WA05NG | NDAR_INVDH29TC6V | NDAR_INVK1WPW1EZ | NDAR_INVRALE29MJ | NDAR_INVXLP913HM |
| NDAR_INV33AC40WZ | NDAR_INV89ZP9REV | NDAR_INVDH2GTB06 | NDAR_INVK1YTJYND | NDAR_INVRAPG65HL | NDAR_INVXLXW4PP4 |
| NDAR_INV33FEYMMB | NDAR_INV8AMD17WG | NDAR_INVDH8W6H22 | NDAR_INVK20NJZWV | NDAR_INVRAR30NKY | NDAR_INVXLYDYTLX |
| NDAR_INV344TNJRC | NDAR_INV8ANDJD6V | NDAR_INVDHCPMWJD | NDAR_INVK21Y8N4U | NDAR_INVRBNRB1DU | NDAR_INVXMFG5XLJ |
| NDAR_INV34BZY5KP | NDAR_INV8AWUPZE7 | NDAR_INVDHDLJ5CV | NDAR_INVK22WXK72 | NDAR_INVRBTUEV72 | NDAR_INVXMJE5DN0 |
| NDAR_INV34HCN2RW | NDAR_INV8B8WV7MT | NDAR_INVDHRT3GB2 | NDAR_INVK2503EYB | NDAR_INVRBUNMARM | NDAR_INVXMMZB7E9 |
| NDAR_INV352NLM92 | NDAR_INV8BMGPD45 | NDAR_INVDJ22WEAT | NDAR_INVK2JE6A93 | NDAR_INVRCFLYCF4 | NDAR_INVXMP1CA5E |
| NDAR_INV352XG4XH | NDAR_INV8BMH9VM4 | NDAR_INVDJ81TV7Z | NDAR_INVK2M95XYW | NDAR_INVRCHKCHLP | NDAR_INVXMWVP3Y9 |
| NDAR_INV35E15ZE0 | NDAR_INV8C2MCZ4K | NDAR_INVDJHUKGLH | NDAR_INVK2VDYAKB | NDAR_INVRCXPE6ZR | NDAR_INVXMYHNHRX |
| NDAR_INV361NRHEX | NDAR_INV8CGB04GK | NDAR_INVDJU1GZNN | NDAR_INVK2YJBHGX | NDAR_INVRD63XMP9 | NDAR_INVXN1RT0M9 |
| NDAR_INV36E4JVZ2 | NDAR_INV8CT52P5H | NDAR_INVDKEXEJKC | NDAR_INVK31MTN10 | NDAR_INVRDXFBTYA | NDAR_INVXN6HMGK8 |
| NDAR_INV36JGB4H6 | NDAR_INV8DRXTTAT | NDAR_INVDLE6LXEP | NDAR_INVK39208D1 | NDAR_INVRE3H89UH | NDAR_INVXNCB4UPY |
| NDAR_INV370C5J23 | NDAR_INV8E028M6F | NDAR_INVDLEMTN1E | NDAR_INVK3GPV04G | NDAR_INVRE4XCDJR | NDAR_INVXNEVU6X0 |
| NDAR_INV37AGV6H4 | NDAR_INV8E2WVMNK | NDAR_INVDLG15XM2 | NDAR_INVK4KJGM46 | NDAR_INVREK6RR1X | NDAR_INVXNJ0GBYC |
| NDAR_INV37DLYV6T | NDAR_INV8E597FU6 | NDAR_INVDLMML76V | NDAR_INVK51MXR7P | NDAR_INVRELT7MMW | NDAR_INVXNKDAEH6 |
| NDAR_INV37KJF13T | NDAR_INV8E5E6YV1 | NDAR_INVDMD17V8N | NDAR_INVK52CCTV1 | NDAR_INVRF0K5LV7 | NDAR_INVXNU4XR8E |
| NDAR_INV37XJVFZN | NDAR_INV8EH16V1R | NDAR_INVDMXMW991 | NDAR_INVK598LFL1 | NDAR_INVRF2AA961 | NDAR_INVXP71CVE9 |
| NDAR_INV37ZMYZNR | NDAR_INV8EM420D1 | NDAR_INVDN11K433 | NDAR_INVK5DJG2BK | NDAR_INVRF70B79M | NDAR_INVXP75417U |
| NDAR_INV3807LYLG | NDAR_INV8EUP2KFK | NDAR_INVDN2W0CYG | NDAR_INVK5P234R5 | NDAR_INVRFFGJ02J | NDAR_INVXPLPX26E |
| NDAR_INV385XPJ9D | NDAR_INV8F1DEP52 | NDAR_INVDNNCWZP6 | NDAR_INVK5UA0808 | NDAR_INVRFLMRCMP | NDAR_INVXRHZDZE8 |
| NDAR_INV38FX2APW | NDAR_INV8FCEHCME | NDAR_INVDP5670V4 | NDAR_INVK638E3TP | NDAR_INVRFNHTDPM | NDAR_INVXRNZ5M92 |
| NDAR_INV38MDKPJC | NDAR_INV8FPU6BRJ | NDAR_INVDPB7N7HA | NDAR_INVK6V3W39C | NDAR_INVRFV3V3DV | NDAR_INVXRWF7AVL |
| NDAR_INV38RT7JJA | NDAR_INV8FYG1NE5 | NDAR_INVDPP9W7AV | NDAR_INVK6W4APWD | NDAR_INVRG22U5T9 | NDAR_INVXT3G7YJR |
| NDAR_INV38T88Z04 | NDAR_INV8GNT6WT8 | NDAR_INVDR3M36J1 | NDAR_INVK6WRX260 | NDAR_INVRGAY5U2D | NDAR_INVXTBCPTH4 |
| NDAR_INV38TUEK9N | NDAR_INV8GP2PFEE | NDAR_INVDT2WNL94 | NDAR_INVK71K7VF1 | NDAR_INVRGF2CX0R | NDAR_INVXTTCDPV5 |
| NDAR_INV395VW31D | NDAR_INV8GWBGTAB | NDAR_INVDT85NUFE | NDAR_INVK71MKKBH | NDAR_INVRGHUCFDR | NDAR_INVXTUP2ECD |
| NDAR_INV396JNWVX | NDAR_INV8GZUK8EV | NDAR_INVDTHJM3Y9 | NDAR_INVK78X1G9D | NDAR_INVRGLBEBD1 | NDAR_INVXU6572VY |
| NDAR_INV39DNG05T | NDAR_INV8H756ZWX | NDAR_INVDTKLRD8X | NDAR_INVK7ZHC09E | NDAR_INVRGVY0VAU | NDAR_INVXU9WL7P7 |
| NDAR_INV39Z1U19N | NDAR_INV8HB05LMR | NDAR_INVDU50FZ05 | NDAR_INVK81PDBDN | NDAR_INVRGY07HCK | NDAR_INVXUAL3R9B |
| NDAR_INV3A81U14B | NDAR_INV8HX2TBDP | NDAR_INVDU5LEWJK | NDAR_INVK832EFEE | NDAR_INVRH6B02GB | NDAR_INVXUJ4X428 |
| NDAR_INV3AD71HE2 | NDAR_INV8J5VU553 | NDAR_INVDUJJ740F | NDAR_INVK8G0M5HW | NDAR_INVRH6ZTTH4 | NDAR_INVXUKGKUMG |
| NDAR_INV3ADKR0X9 | NDAR_INV8JE580G0 | NDAR_INVDUKN0GD9 | NDAR_INVK91CAXRJ | NDAR_INVRH8MPM9J | NDAR_INVXV7ACNJ4 |
| NDAR_INV3ADKZJ2Z | NDAR_INV8JHCHHNT | NDAR_INVDV403EUN | NDAR_INVK9CK3ZHW | NDAR_INVRHG5A1YK | NDAR_INVXV7GLFGG |
| NDAR_INV3AUPJL86 | NDAR_INV8JJXHDK8 | NDAR_INVDVEWNJZ1 | NDAR_INVKA96L5HC | NDAR_INVRHLEYZMW | NDAR_INVXV9T20V8 |
| NDAR_INV3B60KLEU | NDAR_INV8JRW2UVW | NDAR_INVDVJ76ALR | NDAR_INVKACJ255N | NDAR_INVRHX34P95 | NDAR_INVXVDG45XY |
| NDAR_INV3CE271ET | NDAR_INV8JX18G49 | NDAR_INVDVVR7GEL | NDAR_INVKAZ30BTB | NDAR_INVRHZUTPG3 | NDAR_INVXVHRL939 |
| NDAR_INV3CVWRF42 | NDAR_INV8K5LRYHL | NDAR_INVDWJKX2YR | NDAR_INVKB6F3HGV | NDAR_INVRJCEU60H | NDAR_INVXVRX9PPJ |
| NDAR_INV3DMNEA3Z | NDAR_INV8KHWVMVW | NDAR_INVDWKHJ8MG | NDAR_INVKBJJ7TH0 | NDAR_INVRJLKFF0B | NDAR_INVXVT1UD9C |
| NDAR_INV3ERJWDKZ | NDAR_INV8KUKEWXK | NDAR_INVDWP35H7Y | NDAR_INVKC24Z5V5 | NDAR_INVRJTWKG0P | NDAR_INVXW3ZZZXY |
| NDAR_INV3F2UEE7D | NDAR_INV8L43BG60 | NDAR_INVDWTK3PP0 | NDAR_INVKCAUT6AU | NDAR_INVRK3814U8 | NDAR_INVXWKTXRJG |
| NDAR_INV3F5L113U | NDAR_INV8L7MBY64 | NDAR_INVDXVD4UW0 | NDAR_INVKCM3GAKP | NDAR_INVRKD9X23X | NDAR_INVXWN727UB |
| NDAR_INV3F6NJ6WW | NDAR_INV8L88HZVW | NDAR_INVDY0X871X | NDAR_INVKCW9B4TJ | NDAR_INVRKF98ZFJ | NDAR_INVXWW4FAU3 |
| NDAR_INV3FJ6DHVU | NDAR_INV8LBGM6LE | NDAR_INVDYB30L2R | NDAR_INVKCXJ48Z1 | NDAR_INVRL198MFV | NDAR_INVXWZ04XEN |
| NDAR_INV3H19ZCF8 | NDAR_INV8LUWPYZD | NDAR_INVDYNUN0GN | NDAR_INVKDZR86PA | NDAR_INVRLH4F4JA | NDAR_INVXX3MZNV7 |
| NDAR_INV3H81JJ1U | NDAR_INV8M3JENZ9 | NDAR_INVDZ8GELZ6 | NDAR_INVKE2PDM4E | NDAR_INVRLV2KEJP | NDAR_INVXXKJDH17 |
| NDAR_INV3HBNNTXH | NDAR_INV8M5R0YYF | NDAR_INVDZKEBE4K | NDAR_INVKF0KH7ZK | NDAR_INVRMB0B610 | NDAR_INVXXR7E90Z |
| NDAR_INV3HC4V43B | NDAR_INV8M6K3LNP | NDAR_INVDZLD38UM | NDAR_INVKFH1X898 | NDAR_INVRMH1RF1E | NDAR_INVXXZTK34K |
| NDAR_INV3HEHP1P4 | NDAR_INV8M6XN83T | NDAR_INVDZVTE3N0 | NDAR_INVKFJWT11B | NDAR_INVRMNMGK77 | NDAR_INVXYLTMBZ9 |
| NDAR_INV3HFPA2R0 | NDAR_INV8MGGJ4FD | NDAR_INVE0577FC4 | NDAR_INVKFMJ0NH7 | NDAR_INVRMTA5CWA | NDAR_INVXYP99M7T |
| NDAR_INV3HJUHZX1 | NDAR_INV8MJUZ27T | NDAR_INVE06V8Y23 | NDAR_INVKFNA5PDB | NDAR_INVRMTE592D | NDAR_INVXZ3MMX6M |
| NDAR_INV3HMZ7DZZ | NDAR_INV8MKMV27D | NDAR_INVE0AED7HM | NDAR_INVKFNWR98R | NDAR_INVRMTJ44GE | NDAR_INVXZ8BRRCH |
| NDAR_INV3HZG2Y7P | NDAR_INV8MRME9FG | NDAR_INVE0B9A5VN | NDAR_INVKFUK0D3N | NDAR_INVRMU6AKL2 | NDAR_INVXZCJNKR9 |
| NDAR_INV3JNFV6TN | NDAR_INV8NKY4X1D | NDAR_INVE0CL572F | NDAR_INVKGNHNNA3 | NDAR_INVRN93HUJE | NDAR_INVY064R00W |
| NDAR_INV3JUUH0FL | NDAR_INV8NLT77TT | NDAR_INVE0KZKF5V | NDAR_INVKGRR3WRJ | NDAR_INVRNEHTMV4 | NDAR_INVY09XGV2D |
| NDAR_INV3JYXAP1H | NDAR_INV8NRHNVZ9 | NDAR_INVE0MBDEG1 | NDAR_INVKGUR3RYT | NDAR_INVRP0XB0ZR | NDAR_INVY0CRE49N |
| NDAR_INV3KAPX6MY | NDAR_INV8P1H87D1 | NDAR_INVE1MRKJ3N | NDAR_INVKH0DH6EC | NDAR_INVRP3R4YCP | NDAR_INVY0M8DA4H |
| NDAR_INV3KYJ3920 | NDAR_INV8P62FV1D | NDAR_INVE2755CEH | NDAR_INVKJ6VDNZC | NDAR_INVRPY0VAPZ | NDAR_INVY0RZGBNN |
| NDAR_INV3KZATZ7M | NDAR_INV8PAZYD33 | NDAR_INVE2887AGF | NDAR_INVKJCKZPX0 | NDAR_INVRPYKZRFL | NDAR_INVY17ZU37V |
| NDAR_INV3L9YHDDK | NDAR_INV8PFCYLGD | NDAR_INVE2GEK6A2 | NDAR_INVKJCYGTUC | NDAR_INVRR22WRZJ | NDAR_INVY1B9FTLD |
| NDAR_INV3LA2YW3R | NDAR_INV8PM4DHGY | NDAR_INVE2PWTA4F | NDAR_INVKJKYNK5V | NDAR_INVRR7J1YX2 | NDAR_INVY1KBMF25 |
| NDAR_INV3LTP4JU7 | NDAR_INV8PMAFPCV | NDAR_INVE367LYLC | NDAR_INVKJY3WLAB | NDAR_INVRRAW1J1K | NDAR_INVY2B2659F |
| NDAR_INV3M6TK7TT | NDAR_INV8PUU0W5P | NDAR_INVE39X2HV4 | NDAR_INVKK1AVL1G | NDAR_INVRRE6MVYZ | NDAR_INVY2BY2AXF |
| NDAR_INV3MANP3Y8 | NDAR_INV8PVUD11K | NDAR_INVE3GZGT66 | NDAR_INVKK3DUDE5 | NDAR_INVRREA9RBW | NDAR_INVY2DXN8WF |
| NDAR_INV3MJ73KP6 | NDAR_INV8R988ZMR | NDAR_INVE3NPVWW3 | NDAR_INVKK61BBH5 | NDAR_INVRRFZW203 | NDAR_INVY36XFLGJ |
| NDAR_INV3MR1M0V3 | NDAR_INV8RHA34G8 | NDAR_INVE42NY47E | NDAR_INVKK8HT4H8 | NDAR_INVRRR6XT5R | NDAR_INVY38WEZBE |
| NDAR_INV3MTP07E9 | NDAR_INV8RN0AJRP | NDAR_INVE46BJ2YA | NDAR_INVKKTCT4B0 | NDAR_INVRRT6GJ3T | NDAR_INVY39Z6PHB |
| NDAR_INV3MXVVRKB | NDAR_INV8RP64EBL | NDAR_INVE56HZVKV | NDAR_INVKLBUDEGD | NDAR_INVRRTJUY6G | NDAR_INVY3HRTGKA |
| NDAR_INV3N0KGZ5G | NDAR_INV8TJJB3MY | NDAR_INVE5L7WXDY | NDAR_INVKLE3R4XB | NDAR_INVRTD32ZG1 | NDAR_INVY3LBB0K3 |
| NDAR_INV3NABWF15 | NDAR_INV8TJPUWXP | NDAR_INVE5WTBCCC | NDAR_INVKLMJ728E | NDAR_INVRTLY443G | NDAR_INVY3N5BMUP |
| NDAR_INV3NGLDPCG | NDAR_INV8TLTP9PT | NDAR_INVE6D54XED | NDAR_INVKLT2ZLDF | NDAR_INVRU3LYZZF | NDAR_INVY3YXX5T8 |
| NDAR_INV3NT6ML17 | NDAR_INV8TUJVDPE | NDAR_INVE6E13XJZ | NDAR_INVKM0BYRA9 | NDAR_INVRU8N35XR | NDAR_INVY43C1PAY |
| NDAR_INV3P1U2ABW | NDAR_INV8TWGX6LF | NDAR_INVE6KKE4TT | NDAR_INVKM0YCM9R | NDAR_INVRUKL1DUN | NDAR_INVY43H2LJL |
| NDAR_INV3P7CNL7H | NDAR_INV8U17X9M3 | NDAR_INVE736Y9K5 | NDAR_INVKM1TZDEV | NDAR_INVRUPYH7NX | NDAR_INVY463K3XX |
| NDAR_INV3PH12WM5 | NDAR_INV8U909UGM | NDAR_INVE7949ZLU | NDAR_INVKM9M2HZM | NDAR_INVRV0X5P44 | NDAR_INVY4D2ZRMT |
| NDAR_INV3PH5L11X | NDAR_INV8UF58XPK | NDAR_INVE7ZTHWWW | NDAR_INVKMB705H9 | NDAR_INVRW8JB8M2 | NDAR_INVY4DLE2ZX |
| NDAR_INV3RF0BXXZ | NDAR_INV8UV3CABP | NDAR_INVE8DG7RVD | NDAR_INVKN9RD01B | NDAR_INVRWEFC99G | NDAR_INVY4NY9NDV |
| NDAR_INV3RK373VR | NDAR_INV8UVMNBZL | NDAR_INVE8FNCVCL | NDAR_INVKNAZCFHN | NDAR_INVRWZV4MWW | NDAR_INVY4WGB6A3 |
| NDAR_INV3RLHYZEL | NDAR_INV8V5WVRJ3 | NDAR_INVE92HH5DM | NDAR_INVKNBJ47HV | NDAR_INVRXDY2EL7 | NDAR_INVY5CRB0R8 |
| NDAR_INV3RTWYZX5 | NDAR_INV8V8W0LJR | NDAR_INVE9VXBRYU | NDAR_INVKNFUZV4X | NDAR_INVRXN9H1J6 | NDAR_INVY5GHPDX4 |
| NDAR_INV3T4N9EJD | NDAR_INV8W2HU9CN | NDAR_INVEAEC49WA | NDAR_INVKNHZYKRL | NDAR_INVRY4YXW7M | NDAR_INVY5VUA73W |
| NDAR_INV3TBTUYZ5 | NDAR_INV8X0UYC73 | NDAR_INVEAKFA13T | NDAR_INVKNMWKW2X | NDAR_INVRYB7W7HP | NDAR_INVY650YFT4 |
| NDAR_INV3TFY3VZV | NDAR_INV8X4L319G | NDAR_INVEANH4HXE | NDAR_INVKPH9VE20 | NDAR_INVRYT5RRUM | NDAR_INVY6KXTXY4 |
| NDAR_INV3TG667EC | NDAR_INV8X6CPV8N | NDAR_INVEAP0TNV8 | NDAR_INVKPLUBC73 | NDAR_INVRZ3AMJJG | NDAR_INVY7EU2VZ6 |
| NDAR_INV3TKL2EYB | NDAR_INV8XDHCHEA | NDAR_INVEB844BJA | NDAR_INVKR1PVY4C | NDAR_INVRZBKNZ42 | NDAR_INVY81M9FZH |
| NDAR_INV3TUE7DV5 | NDAR_INV8XTC7XWY | NDAR_INVEBVXV8NL | NDAR_INVKR3R305M | NDAR_INVRZL7PGK1 | NDAR_INVY8CBY4HN |
| NDAR_INV3UTXWW38 | NDAR_INV8Y6RVMNM | NDAR_INVEC4FTVA6 | NDAR_INVKT7E0NA1 | NDAR_INVRZTG711F | NDAR_INVY8CPCJJ3 |
| NDAR_INV3UUD5J8C | NDAR_INV8Y8A8NU0 | NDAR_INVECDXKKY4 | NDAR_INVKT8MVW3Z | NDAR_INVT07JTPKT | NDAR_INVY8P9LNN8 |
| NDAR_INV3WDYMBWU | NDAR_INV8YGWRPZ8 | NDAR_INVECNLRY0U | NDAR_INVKTJFT8RF | NDAR_INVT09Y5F0B | NDAR_INVY956TX1N |
| NDAR_INV3WEDXU2N | NDAR_INV8YKR9JAK | NDAR_INVECTME361 | NDAR_INVKTN3FFWD | NDAR_INVT15ZEHBH | NDAR_INVY9YA660C |
| NDAR_INV3WH5A5LB | NDAR_INV8YXW9AD2 | NDAR_INVED2RMTP1 | NDAR_INVKTZJFPCA | NDAR_INVT161L03G | NDAR_INVYA69C55Y |
| NDAR_INV3WT1TK9L | NDAR_INV8Z3UW49L | NDAR_INVED2XF3C4 | NDAR_INVKU1J9JFE | NDAR_INVT1RR6C3X | NDAR_INVYAFVGGKU |
| NDAR_INV3WXXPJKW | NDAR_INV8Z59C4WA | NDAR_INVED4GJ51W | NDAR_INVKUTBPBRE | NDAR_INVT1VTW1HM | NDAR_INVYAKZW65A |
| NDAR_INV3X1TYL13 | NDAR_INV8ZT3RL78 | NDAR_INVEDALBX5M | NDAR_INVKV1CFR99 | NDAR_INVT21R0YG4 | NDAR_INVYAXN63F8 |
| NDAR_INV3XM1RJF0 | NDAR_INV9053L040 | NDAR_INVEDE0TXYZ | NDAR_INVKV39ALMD | NDAR_INVT226J5Y3 | NDAR_INVYB10EE8B |
| NDAR_INV3XNH8EYM | NDAR_INV905XNJK8 | NDAR_INVEDF5950W | NDAR_INVKVBV80LE | NDAR_INVT23YJZWZ | NDAR_INVYBC941EU |
| NDAR_INV3XNNBDPH | NDAR_INV908NEAJ2 | NDAR_INVEDLY2KYA | NDAR_INVKVGJL857 | NDAR_INVT2CAXTA1 | NDAR_INVYBJPP4VV |
| NDAR_INV3Y3HGYYU | NDAR_INV90RDFNXK | NDAR_INVEE52ZTTG | NDAR_INVKVMU7J2K | NDAR_INVT2GU26AW | NDAR_INVYBPWDCF6 |
| NDAR_INV3YJ5XDU0 | NDAR_INV90T8PKLB | NDAR_INVEERV77GP | NDAR_INVKWG00F0U | NDAR_INVT2RKLJ1J | NDAR_INVYC3MCRAY |
| NDAR_INV3YJA47GG | NDAR_INV910NJYX0 | NDAR_INVEEY2XYCA | NDAR_INVKX7Y3XLZ | NDAR_INVT2YYABW3 | NDAR_INVYC5NU1KR |
| NDAR_INV3YRA5895 | NDAR_INV915YKBPY | NDAR_INVEF02CVNX | NDAR_INVKX9VFDRV | NDAR_INVT3366FHE | NDAR_INVYCCKAV9K |
| NDAR_INV3YVFTTE6 | NDAR_INV91BAHLL2 | NDAR_INVEFAV5D00 | NDAR_INVKXCAUBG6 | NDAR_INVT4BR7L9V | NDAR_INVYCMGF947 |
| NDAR_INV3YXV3Y6W | NDAR_INV91NNHKF0 | NDAR_INVEFCVZYTR | NDAR_INVKXEBWC6V | NDAR_INVT4M6PYF4 | NDAR_INVYCVPJ3MW |
| NDAR_INV3ZC3XKET | NDAR_INV91X6JZ76 | NDAR_INVEFEM89HR | NDAR_INVKXLA75F1 | NDAR_INVT58P12VM | NDAR_INVYD28H9BD |
| NDAR_INV3ZCVTCG4 | NDAR_INV91ZBKM4B | NDAR_INVEG7V5UN3 | NDAR_INVKXUNRZDG | NDAR_INVT5B642W6 | NDAR_INVYD98TZ0C |
| NDAR_INV3ZPCUAK0 | NDAR_INV920MEG9V | NDAR_INVEGCADPBF | NDAR_INVKXZGFV4A | NDAR_INVT5EXYEGX | NDAR_INVYDF2TPMK |
| NDAR_INV3ZPDZ9KU | NDAR_INV9245VEY5 | NDAR_INVEGCJDH40 | NDAR_INVKY6C0Z8R | NDAR_INVT5RV9MLG | NDAR_INVYDMW8MYL |
| NDAR_INV3ZRYG6UB | NDAR_INV925PNR1C | NDAR_INVEGFEVU9N | NDAR_INVKYCN6AYE | NDAR_INVT5VEMC05 | NDAR_INVYE30YAL9 |
| NDAR_INV3ZT6V44L | NDAR_INV92WZKT4Y | NDAR_INVEGHAHA0D | NDAR_INVKYJCM061 | NDAR_INVT65K9GLZ | NDAR_INVYE99F6V6 |
| NDAR_INV3ZX15022 | NDAR_INV92Z89C3Z | NDAR_INVEGRH63PV | NDAR_INVKYXDJC5Z | NDAR_INVT6EC6R57 | NDAR_INVYEEC34R8 |
| NDAR_INV403JXRX5 | NDAR_INV92ZMA6L7 | NDAR_INVEH3T61EE | NDAR_INVKZNB0MYE | NDAR_INVT6GGB7YF | NDAR_INVYEFA2634 |
| NDAR_INV40J2BT3D | NDAR_INV930295U4 | NDAR_INVEHP8VX1B | NDAR_INVKZT2PNJK | NDAR_INVT6RG755A | NDAR_INVYEH878VD |
| NDAR_INV40NNEMXD | NDAR_INV9316NFE7 | NDAR_INVEJ1NL7U8 | NDAR_INVL07PRMYX | NDAR_INVT6XZEJHF | NDAR_INVYEKG7D1H |
| NDAR_INV40UDB664 | NDAR_INV93CTR3FD | NDAR_INVEJ2BUCAH | NDAR_INVL08R9YMD | NDAR_INVT6Z99TKW | NDAR_INVYEM9LW83 |
| NDAR_INV40YFT2B2 | NDAR_INV93J5H7BU | NDAR_INVEJ8E4U10 | NDAR_INVL0YU2ZAP | NDAR_INVT7KRTJPP | NDAR_INVYEMHGFJF |
| NDAR_INV414B0X0A | NDAR_INV93W1HHJN | NDAR_INVEJJGPBL3 | NDAR_INVL18N9A9R | NDAR_INVT83EVU05 | NDAR_INVYEZ6T0J8 |
| NDAR_INV41A6MY1V | NDAR_INV93WX6071 | NDAR_INVEJXM20D6 | NDAR_INVL1GA12Y1 | NDAR_INVT8FJ7FK6 | NDAR_INVYFEGLL2B |
| NDAR_INV41L5KPJC | NDAR_INV94DY9KKD | NDAR_INVEKJJFW9J | NDAR_INVL1PVW5B7 | NDAR_INVT8KRKTVN | NDAR_INVYFGJBRCE |
| NDAR_INV41RVJVER | NDAR_INV94LUVVX8 | NDAR_INVEKL2JGYR | NDAR_INVL1RVKM6B | NDAR_INVT984W7CU | NDAR_INVYFT16CPZ |
| NDAR_INV41U1CNA9 | NDAR_INV94NCJR76 | NDAR_INVEKRGXJZ0 | NDAR_INVL1UHN7LG | NDAR_INVT9E1CUGM | NDAR_INVYFW25B6F |
| NDAR_INV4206Z9NR | NDAR_INV94TFREHY | NDAR_INVEKYG0E33 | NDAR_INVL1XJPZEL | NDAR_INVT9F6AYDG | NDAR_INVYGD67PV4 |
| NDAR_INV4208ZTJH | NDAR_INV94V97129 | NDAR_INVEKZEA71F | NDAR_INVL2FYHWYV | NDAR_INVT9HJ5VP1 | NDAR_INVYH3BPP7A |
| NDAR_INV422PKRTL | NDAR_INV956TANLH | NDAR_INVEL21AUAJ | NDAR_INVL3258K1X | NDAR_INVT9UU6CM8 | NDAR_INVYH4D9KHP |
| NDAR_INV423DABE7 | NDAR_INV959DHBNF | NDAR_INVEL8YUZKU | NDAR_INVL3DBH8WC | NDAR_INVT9YPUK1P | NDAR_INVYH8W7XH2 |
| NDAR_INV4275X676 | NDAR_INV95L3764E | NDAR_INVELJ01PAA | NDAR_INVL3E24BP4 | NDAR_INVTABF5AC1 | NDAR_INVYHJA3NXM |
| NDAR_INV42EDG566 | NDAR_INV95WR5KVA | NDAR_INVELW92005 | NDAR_INVL42FE3NR | NDAR_INVTADXZB2Y | NDAR_INVYHMMV4AC |
| NDAR_INV42ELB372 | NDAR_INV96536WUJ | NDAR_INVEN1P5RFL | NDAR_INVL453XJAG | NDAR_INVTAMN62RZ | NDAR_INVYHRM6MHF |
| NDAR_INV42J1GB3Y | NDAR_INV968XN9T9 | NDAR_INVEP0DL4X4 | NDAR_INVL456T3RZ | NDAR_INVTANY2ML3 | NDAR_INVYJ7PYHV7 |
| NDAR_INV42LK7GJJ | NDAR_INV96G7MJ87 | NDAR_INVEP4T9K93 | NDAR_INVL46T49EG | NDAR_INVTAX3MN8C | NDAR_INVYJA9K9F8 |
| NDAR_INV43DEA5HT | NDAR_INV96X5ZCCH | NDAR_INVEP6DZX2M | NDAR_INVL4UEG9G0 | NDAR_INVTAY698CJ | NDAR_INVYJAFABD8 |
| NDAR_INV43VJ4GZ1 | NDAR_INV97K3KGF4 | NDAR_INVEPEE5V5J | NDAR_INVL53XA17C | NDAR_INVTB68WR1L | NDAR_INVYJHDGNJN |
| NDAR_INV43YWL3H0 | NDAR_INV97RLXMF9 | NDAR_INVEPHNCTV0 | NDAR_INVL542JFCM | NDAR_INVTB7KL2UL | NDAR_INVYJL7Y7CB |
| NDAR_INV44K29WLN | NDAR_INV97YHGYC9 | NDAR_INVEPMR7U1F | NDAR_INVL5AY1R6V | NDAR_INVTBJX7XV9 | NDAR_INVYK2J75TH |
| NDAR_INV44VXW7YA | NDAR_INV98096D02 | NDAR_INVEPVBPNVU | NDAR_INVL5GHARC8 | NDAR_INVTBTBAM4J | NDAR_INVYK5U2D70 |
| NDAR_INV44WLHHC1 | NDAR_INV98E4JFTY | NDAR_INVERNFXT89 | NDAR_INVL5RBR153 | NDAR_INVTC53X138 | NDAR_INVYKFPYD54 |
| NDAR_INV455D0ZHD | NDAR_INV9924G4W9 | NDAR_INVERWG31K7 | NDAR_INVL5TG7F2T | NDAR_INVTCAFT1FE | NDAR_INVYKVPYY2M |
| NDAR_INV459DR5EV | NDAR_INV99BP99WU | NDAR_INVET1RUD2W | NDAR_INVL5YY3CJB | NDAR_INVTCEFC244 | NDAR_INVYL2LMJ28 |
| NDAR_INV45BG08PF | NDAR_INV99TVX9G8 | NDAR_INVET4W53Z8 | NDAR_INVL63XHP8H | NDAR_INVTCJV2RMX | NDAR_INVYL42LJHV |
| NDAR_INV45WKYG93 | NDAR_INV9A111GA5 | NDAR_INVETDKN8PJ | NDAR_INVL6J5NEVL | NDAR_INVTCUKBDE0 | NDAR_INVYLC0XGH4 |
| NDAR_INV46VFKP3F | NDAR_INV9A4WYDA8 | NDAR_INVETF368ZG | NDAR_INVL6ZD3MC6 | NDAR_INVTCVY0CYH | NDAR_INVYLKWWGJ2 |
| NDAR_INV474A4X05 | NDAR_INV9AD6NWVN | NDAR_INVETNF2G44 | NDAR_INVL7G860LK | NDAR_INVTD0D86H6 | NDAR_INVYLMRXPYA |
| NDAR_INV47N6G712 | NDAR_INV9AEE483X | NDAR_INVETWT6P43 | NDAR_INVL7XCKG7W | NDAR_INVTDCYY0EZ | NDAR_INVYLNRHT83 |
| NDAR_INV47UWAATW | NDAR_INV9AHK0Y6D | NDAR_INVEU0LET8P | NDAR_INVL7ZXH7GM | NDAR_INVTDK7YAGN | NDAR_INVYM6A1LU9 |
| NDAR_INV481L0PYY | NDAR_INV9AJ433R7 | NDAR_INVEU57U8JH | NDAR_INVL8EJK2VY | NDAR_INVTDXY0L82 | NDAR_INVYMPEHMMT |
| NDAR_INV486GVBCD | NDAR_INV9AMTA922 | NDAR_INVEUR2B638 | NDAR_INVL8W0WHE1 | NDAR_INVTEB0VDTW | NDAR_INVYN247XZX |
| NDAR_INV486UUF4D | NDAR_INV9AULPA6C | NDAR_INVEV2TLTFZ | NDAR_INVL8XFXVEJ | NDAR_INVTEB9HYMX | NDAR_INVYN5MT0RT |
| NDAR_INV4878W3DV | NDAR_INV9AWJE5BU | NDAR_INVEVC26KZ4 | NDAR_INVL8XGD85G | NDAR_INVTEGHFYFP | NDAR_INVYN5P4E5V |
| NDAR_INV48EYUFJ4 | NDAR_INV9B14E538 | NDAR_INVEVFG8LC1 | NDAR_INVL96K1YR0 | NDAR_INVTELJP2LE | NDAR_INVYNANG2B0 |
| NDAR_INV48JAWLFK | NDAR_INV9B3TN6RL | NDAR_INVEVN2CFJL | NDAR_INVL9L2X47R | NDAR_INVTEZ4278H | NDAR_INVYNCHCTAD |
| NDAR_INV48LY4MLX | NDAR_INV9BH45R0D | NDAR_INVEW0CN0CD | NDAR_INVL9NJJJBZ | NDAR_INVTEZKLFY3 | NDAR_INVYNRJ8UU6 |
| NDAR_INV48RC3H1N | NDAR_INV9BV5RW8J | NDAR_INVEW9ZPKZK | NDAR_INVL9NUBDAN | NDAR_INVTFE6R22P | NDAR_INVYPRNYTTL |
| NDAR_INV493AHEA8 | NDAR_INV9C4CCRWN | NDAR_INVEWDP96RH | NDAR_INVLABCHP1W | NDAR_INVTFED6EEK | NDAR_INVYPWGL6HJ |
| NDAR_INV49B5ZDR2 | NDAR_INV9DWM4Z1U | NDAR_INVEWMZTM2M | NDAR_INVLALRAZCB | NDAR_INVTFFA4ZV9 | NDAR_INVYRBADWT5 |
| NDAR_INV49D4EDYG | NDAR_INV9EC8VLZC | NDAR_INVEX51JT1N | NDAR_INVLB0H15T3 | NDAR_INVTFJ3K89H | NDAR_INVYRVP8LV1 |
| NDAR_INV49EG90M3 | NDAR_INV9EG02533 | NDAR_INVEXB1B45G | NDAR_INVLB5D5679 | NDAR_INVTFPAU4FF | NDAR_INVYT5WUN07 |
| NDAR_INV49PA9KKY | NDAR_INV9EM32PUW | NDAR_INVEY4BB6R7 | NDAR_INVLBAKFHGK | NDAR_INVTFU3B4T0 | NDAR_INVYTH97JCP |
| NDAR_INV49YTZ5Z4 | NDAR_INV9EVRB30H | NDAR_INVEYANDC02 | NDAR_INVLBWER7ME | NDAR_INVTFV0Y41D | NDAR_INVYTJ92RU3 |
| NDAR_INV4A443KDZ | NDAR_INV9EZZCWGY | NDAR_INVEYXZ3PDP | NDAR_INVLBXNVNEW | NDAR_INVTGBMDN8N | NDAR_INVYTKHPZDU |
| NDAR_INV4AP9GM2Z | NDAR_INV9F4G1M9E | NDAR_INVEZHEHCR4 | NDAR_INVLC2XNHVP | NDAR_INVTH6DHG5Y | NDAR_INVYTMYEX1N |
| NDAR_INV4AY58X03 | NDAR_INV9FNUYHC2 | NDAR_INVEZR60E9U | NDAR_INVLC488VTJ | NDAR_INVTHZ2FDT4 | NDAR_INVYU20X3EV |
| NDAR_INV4B1YV01D | NDAR_INV9G3DJ8GF | NDAR_INVEZXL2KXT | NDAR_INVLC4DG7YK | NDAR_INVTJVE3G6N | NDAR_INVYULP6MEY |
| NDAR_INV4BA28Y80 | NDAR_INV9G62YACW | NDAR_INVF00P4A2L | NDAR_INVLCMU795T | NDAR_INVTJZ4N3G9 | NDAR_INVYUPPEV2G |
| NDAR_INV4BEUPF9G | NDAR_INV9GDMLH7C | NDAR_INVF039FKEV | NDAR_INVLCNN89TZ | NDAR_INVTK4DJ93H | NDAR_INVYVJ0JJNF |
| NDAR_INV4BFTB73U | NDAR_INV9GWM2HBT | NDAR_INVF0C17HWX | NDAR_INVLCX3T8HU | NDAR_INVTK6XY92L | NDAR_INVYVVMMD41 |
| NDAR_INV4BK6Y7VR | NDAR_INV9HP92RX0 | NDAR_INVF0C1XLZD | NDAR_INVLCXJ9Y7X | NDAR_INVTKENUL3C | NDAR_INVYW0U0JWC |
| NDAR_INV4BKN34GB | NDAR_INV9HR4XXJ2 | NDAR_INVF0G439YW | NDAR_INVLDA3MRNZ | NDAR_INVTKLRCCP0 | NDAR_INVYW440F27 |
| NDAR_INV4BU3CBM5 | NDAR_INV9HVP5GZ1 | NDAR_INVF0XCUZN6 | NDAR_INVLDDZENEG | NDAR_INVTKXWJF97 | NDAR_INVYWZBJ786 |
| NDAR_INV4C616XF8 | NDAR_INV9JCYGTV3 | NDAR_INVF0ZZ96XL | NDAR_INVLDH3YU2R | NDAR_INVTL3FVU14 | NDAR_INVYX6643N8 |
| NDAR_INV4C8YJ9BG | NDAR_INV9JLEZ6WE | NDAR_INVF10GU9K6 | NDAR_INVLDP9RT86 | NDAR_INVTL570LHZ | NDAR_INVYXATK265 |
| NDAR_INV4CFUY1KU | NDAR_INV9K086XZF | NDAR_INVF19EHX8V | NDAR_INVLE8DHVG1 | NDAR_INVTLBZ1JAC | NDAR_INVYXRGTMYM |
| NDAR_INV4CKYEHYL | NDAR_INV9K1GRJNJ | NDAR_INVF19VWPA1 | NDAR_INVLEF14KHY | NDAR_INVTLEM4F9A | NDAR_INVYYFU8FM4 |
| NDAR_INV4CLN1AC5 | NDAR_INV9K39VXKE | NDAR_INVF1B8U3G5 | NDAR_INVLEND5NH4 | NDAR_INVTM382X5A | NDAR_INVYYTGPBJA |
| NDAR_INV4CNTJPDV | NDAR_INV9K46ANFP | NDAR_INVF1T3HWCV | NDAR_INVLF1ZAD47 | NDAR_INVTM4YT5UD | NDAR_INVYZ0U1DGC |
| NDAR_INV4D81LEVG | NDAR_INV9KCVTWFZ | NDAR_INVF1XKPBT6 | NDAR_INVLFE6F5NR | NDAR_INVTM88BLXR | NDAR_INVYZ353ZBM |
| NDAR_INV4DVGGJE9 | NDAR_INV9KT9V114 | NDAR_INVF2BU99TZ | NDAR_INVLG1ZU0EJ | NDAR_INVTMFT5TJF | NDAR_INVYZFMM6MW |
| NDAR_INV4E0AG51B | NDAR_INV9KTD8UK1 | NDAR_INVF2PT8GN5 | NDAR_INVLG9JZFNH | NDAR_INVTMXKEAP2 | NDAR_INVYZJRWG4A |
| NDAR_INV4EJ61L2C | NDAR_INV9LL2UB0W | NDAR_INVF3RDG1N2 | NDAR_INVLGHA598L | NDAR_INVTN9RVYB1 | NDAR_INVYZURN14T |
| NDAR_INV4ER765J7 | NDAR_INV9M8UXK0A | NDAR_INVF3Y1JRRP | NDAR_INVLH56Y94B | NDAR_INVTNAZAW46 | NDAR_INVZ04GJXZA |
| NDAR_INV4EXN0D8M | NDAR_INV9M8W1495 | NDAR_INVF3Z2K76G | NDAR_INVLHGW76WM | NDAR_INVTNGA8MPC | NDAR_INVZ0A9V1XA |
| NDAR_INV4EY631JB | NDAR_INV9MDFD681 | NDAR_INVF4HCTG43 | NDAR_INVLHZWGFNU | NDAR_INVTNKV2H46 | NDAR_INVZ0LTAG73 |
| NDAR_INV4FBVLRNU | NDAR_INV9MEZA5H2 | NDAR_INVF4PLLUF8 | NDAR_INVLJ74B5M8 | NDAR_INVTNR04C9A | NDAR_INVZ0MP06TT |
| NDAR_INV4FMVZLLG | NDAR_INV9MF93B7R | NDAR_INVF58CLY07 | NDAR_INVLJ8VU64E | NDAR_INVTNVU9DYV | NDAR_INVZ0MXU52L |
| NDAR_INV4FR29VNY | NDAR_INV9N7VAA7U | NDAR_INVF5AE11V8 | NDAR_INVLJJVHGJF | NDAR_INVTP6W072A | NDAR_INVZ0NPTXMB |
| NDAR_INV4FRT1ZYZ | NDAR_INV9N895VTP | NDAR_INVF5G6PA74 | NDAR_INVLJZ1B688 | NDAR_INVTR6TVE72 | NDAR_INVZ0TL0G08 |
| NDAR_INV4FUXW9U4 | NDAR_INV9NFYJG06 | NDAR_INVF5GC1Z80 | NDAR_INVLK2RXTA3 | NDAR_INVTR74D6YC | NDAR_INVZ0XNHVW8 |
| NDAR_INV4G3XF9GX | NDAR_INV9NNKVUGY | NDAR_INVF61FJ1XR | NDAR_INVLK2ZABWM | NDAR_INVTRFMWCAR | NDAR_INVZ174YN3F |
| NDAR_INV4GA9CN7W | NDAR_INV9NXP23VX | NDAR_INVF61PL8NR | NDAR_INVLKGBPW16 | NDAR_INVTRG5GX9T | NDAR_INVZ1A6W085 |
| NDAR_INV4GHXF68C | NDAR_INV9PTH3VTR | NDAR_INVF6553H6E | NDAR_INVLKNX6HF1 | NDAR_INVTT3RTWGF | NDAR_INVZ1MCJXCG |
| NDAR_INV4H9VPPWC | NDAR_INV9PVR76W7 | NDAR_INVF6784XR4 | NDAR_INVLKWJTNDZ | NDAR_INVTTFR6F0H | NDAR_INVZ1VBM6Y7 |
| NDAR_INV4HL4ATJ8 | NDAR_INV9R23N8LH | NDAR_INVF68FFNZG | NDAR_INVLL4RJXKW | NDAR_INVTTVYX9U5 | NDAR_INVZ24E0KC2 |
| NDAR_INV4HX3418K | NDAR_INV9R3YVGD6 | NDAR_INVF6BJCNW6 | NDAR_INVLLUDJYK1 | NDAR_INVTTY8HRTG | NDAR_INVZ2BXG0C8 |
| NDAR_INV4J7L4Y8X | NDAR_INV9R5J1TC2 | NDAR_INVF72J4L68 | NDAR_INVLLUH8L20 | NDAR_INVTUE4K1JE | NDAR_INVZ3XXUKWN |
| NDAR_INV4J8HX541 | NDAR_INV9TCELHYE | NDAR_INVF76ZR3W2 | NDAR_INVLM20BZVZ | NDAR_INVTV25YAN3 | NDAR_INVZ49U1FM4 |
| NDAR_INV4JAZBMAX | NDAR_INV9TE5V8TH | NDAR_INVF7AUUWC6 | NDAR_INVLM6JRR6C | NDAR_INVTV4KFLNU | NDAR_INVZ4KYLWJA |
| NDAR_INV4JD9NHNZ | NDAR_INV9U2F5BG0 | NDAR_INVF7RLL816 | NDAR_INVLM81WCFX | NDAR_INVTV7E93CF | NDAR_INVZ4LY1E6P |
| NDAR_INV4JE67DZA | NDAR_INV9U3NYGX5 | NDAR_INVF86XUHXD | NDAR_INVLMAAJ8JJ | NDAR_INVTX0U3YDP | NDAR_INVZ4YHXYLW |
| NDAR_INV4JNMXAVB | NDAR_INV9UL0YHV1 | NDAR_INVF8GWYLW5 | NDAR_INVLMY0XZB5 | NDAR_INVTX64M9AF | NDAR_INVZ547MN37 |
| NDAR_INV4JRV7N68 | NDAR_INV9UUFK2MY | NDAR_INVF8T2UPP4 | NDAR_INVLMZN1PFG | NDAR_INVTXCRCEXZ | NDAR_INVZ5DDK4LH |
| NDAR_INV4K2HKN0K | NDAR_INV9UW3YHRB | NDAR_INVF8WF9476 | NDAR_INVLN3392MM | NDAR_INVTXK16WY8 | NDAR_INVZ5G203VB |
| NDAR_INV4KBPCMNW | NDAR_INV9V4WNUEA | NDAR_INVF8Z5L5BK | NDAR_INVLN820KH9 | NDAR_INVTXTP560G | NDAR_INVZ62Y3LZB |
| NDAR_INV4KRWWP18 | NDAR_INV9V77D3CT | NDAR_INVF9053E6V | NDAR_INVLN9ZEHNH | NDAR_INVTY4J79EA | NDAR_INVZ64AP7YC |
| NDAR_INV4L1WDFJ7 | NDAR_INV9VGLAX8E | NDAR_INVF9KVX7JE | NDAR_INVLNA2KYAP | NDAR_INVTY7NL56R | NDAR_INVZ694ZZUM |
| NDAR_INV4L9UBRH1 | NDAR_INV9W62GG8X | NDAR_INVF9LU8GW8 | NDAR_INVLNA4879N | NDAR_INVTYTK28UK | NDAR_INVZ6950R7M |
| NDAR_INV4LVCVDEC | NDAR_INV9WFYVL1U | NDAR_INVF9XMG0N0 | NDAR_INVLNF7Y6BR | NDAR_INVTYZ21CK1 | NDAR_INVZ6FGXNPH |
| NDAR_INV4M8H34KB | NDAR_INV9WJ1WBFC | NDAR_INVFA7TAR0L | NDAR_INVLNN6NNXL | NDAR_INVTZ42813G | NDAR_INVZ7U9A2DX |
| NDAR_INV4MPM83XL | NDAR_INV9WT4F32B | NDAR_INVFAFNLCWD | NDAR_INVLNP634D0 | NDAR_INVTZ8JEUMW | NDAR_INVZ85UEYE5 |
| NDAR_INV4N5LBL4W | NDAR_INV9X1R94EZ | NDAR_INVFAUYWJ39 | NDAR_INVLPB8G89P | NDAR_INVTZJ1K01G | NDAR_INVZ8JNGWDY |
| NDAR_INV4N732ND8 | NDAR_INV9X7P9U2G | NDAR_INVFAYT4PA6 | NDAR_INVLR0B3GA6 | NDAR_INVTZUEJ54R | NDAR_INVZ8JVVCW2 |
| NDAR_INV4N77M6YN | NDAR_INV9XA5VMF4 | NDAR_INVFAYYH7CJ | NDAR_INVLR6BN87U | NDAR_INVTZUFYJ76 | NDAR_INVZ8P3EEK3 |
| NDAR_INV4NKVRBAJ | NDAR_INV9Y0DDKT2 | NDAR_INVFB2YC0F5 | NDAR_INVLRAEA45M | NDAR_INVU05WUH4L | NDAR_INVZ8U8TUHJ |
| NDAR_INV4NVY5A41 | NDAR_INV9YBX357L | NDAR_INVFB646F8R | NDAR_INVLTDF7P86 | NDAR_INVU0EVP5JM | NDAR_INVZ95YA2ZB |
| NDAR_INV4NZP000X | NDAR_INV9YG9PWZ2 | NDAR_INVFB9M4ZBX | NDAR_INVLTJFH518 | NDAR_INVU0T6GWNN | NDAR_INVZ96BPL0Y |
| NDAR_INV4P3K4E3R | NDAR_INV9YZV41AE | NDAR_INVFBD5A7P9 | NDAR_INVLU2M4RX1 | NDAR_INVU102M8W0 | NDAR_INVZ9CR6U8L |
| NDAR_INV4P46R5XT | NDAR_INV9Z0LE0BL | NDAR_INVFC3UW2LX | NDAR_INVLUCM81R3 | NDAR_INVU11NYL5H | NDAR_INVZB36FKYX |
| NDAR_INV4PERLVAB | NDAR_INV9Z6E4FXG | NDAR_INVFCE2XV3L | NDAR_INVLUDF8E8N | NDAR_INVU14LH8MY | NDAR_INVZBAL57JN |
| NDAR_INV4PNZ397L | NDAR_INV9ZDG7BT5 | NDAR_INVFCN9U0EN | NDAR_INVLVD0W18X | NDAR_INVU15L4UHW | NDAR_INVZBLU9Z3A |
| NDAR_INV4R6E4A06 | NDAR_INV9ZLX5XU2 | NDAR_INVFD0FRKNT | NDAR_INVLVER5P67 | NDAR_INVU1GL60CG | NDAR_INVZCA261ZR |
| NDAR_INV4R7NANWE | NDAR_INV9ZNYBPMF | NDAR_INVFD5FBUC7 | NDAR_INVLVLHRL2N | NDAR_INVU1MFZFX8 | NDAR_INVZCZPNBKW |
| NDAR_INV4R9BND6G | NDAR_INV9ZTXVA4J | NDAR_INVFDUYJP8Z | NDAR_INVLVT4DDY4 | NDAR_INVU1XPGWVY | NDAR_INVZD57Y0V7 |
| NDAR_INV4RFZF7PZ | NDAR_INVA05T670W | NDAR_INVFE2BKCND | NDAR_INVLVW6C8DX | NDAR_INVU2JURFC1 | NDAR_INVZDLAWZZL |
| NDAR_INV4RX5LRX2 | NDAR_INVA0DJGMNG | NDAR_INVFE5VTN03 | NDAR_INVLW72174Z | NDAR_INVU2UT8NAW | NDAR_INVZDYWHP34 |
| NDAR_INV4TAHM74X | NDAR_INVA0NWYU17 | NDAR_INVFE9E90ZH | NDAR_INVLW9XT5P3 | NDAR_INVU325J0ED | NDAR_INVZEB043HN |
| NDAR_INV4TB7WE3E | NDAR_INVA0RKMN4A | NDAR_INVFF1W5WM0 | NDAR_INVLWH9WH49 | NDAR_INVU38RU1KR | NDAR_INVZEJE1N0G |
| NDAR_INV4TG8X5LW | NDAR_INVA0V2Y3DD | NDAR_INVFFG2LPV0 | NDAR_INVLWR6LC0N | NDAR_INVU3A66CY9 | NDAR_INVZEL29M7N |
| NDAR_INV4TL31WGV | NDAR_INVA12JUH5Z | NDAR_INVFFK2LWBF | NDAR_INVLWVFT8R4 | NDAR_INVU3J6ZW8Z | NDAR_INVZEYJBE3G |
| NDAR_INV4UCBM11Y | NDAR_INVA1A1R65H | NDAR_INVFH0UN282 | NDAR_INVLXAZHZFG | NDAR_INVU3KXEXJ2 | NDAR_INVZF3HPJFM |
| NDAR_INV4UHNX2WX | NDAR_INVA1CW8FGN | NDAR_INVFH4MDTK5 | NDAR_INVLXEAXZ7M | NDAR_INVU3W1437G | NDAR_INVZF3VJWAN |
| NDAR_INV4UPKY2RA | NDAR_INVA1KXWA2W | NDAR_INVFH79L58P | NDAR_INVLXWDWVR4 | NDAR_INVU3ZHJV5Z | NDAR_INVZF8VDCAF |
| NDAR_INV4UYK50KN | NDAR_INVA21KBMCG | NDAR_INVFHBXTPK5 | NDAR_INVLYKAB1UX | NDAR_INVU46YJV27 | NDAR_INVZFGU643V |
| NDAR_INV4V5RG3KP | NDAR_INVA2D0XGJC | NDAR_INVFHK5JU5E | NDAR_INVLZ0K08DY | NDAR_INVU4B58TJY | NDAR_INVZGT6YAX7 |
| NDAR_INV4VUZUGXU | NDAR_INVA2HTKAHY | NDAR_INVFHNEW93L | NDAR_INVLZBR5BLJ | NDAR_INVU4BNFAZ9 | NDAR_INVZHCK39Y1 |
| NDAR_INV4VVRLBUW | NDAR_INVA2JU9A24 | NDAR_INVFJ2WR5PF | NDAR_INVLZE790N4 | NDAR_INVU4LJD50G | NDAR_INVZHF0XGK3 |
| NDAR_INV4VXP5P0U | NDAR_INVA31C7WYJ | NDAR_INVFJAEBAJX | NDAR_INVLZHG1ZK6 | NDAR_INVU5MPKLBP | NDAR_INVZHZZXCM0 |
| NDAR_INV4W3GW9WL | NDAR_INVA36UUP5T | NDAR_INVFJANMBLW | NDAR_INVLZHGWH8V | NDAR_INVU5U9EYAX | NDAR_INVZJ1WJ57J |
| NDAR_INV4WE1GJN2 | NDAR_INVA3CCJD5V | NDAR_INVFJC18PGG | NDAR_INVLZRHC6A5 | NDAR_INVU5V3JUB4 | NDAR_INVZJ7AEU7T |
| NDAR_INV4X3B9V7E | NDAR_INVA3JTEY6X | NDAR_INVFJH3K335 | NDAR_INVM00MPW0G | NDAR_INVU65A7DKA | NDAR_INVZJKN66UT |
| NDAR_INV4X8Z8RG2 | NDAR_INVA3VX7WRD | NDAR_INVFJJPAA2A | NDAR_INVM02A1YVT | NDAR_INVU67FAKAG | NDAR_INVZJM8LDU8 |
| NDAR_INV4X9MDVXC | NDAR_INVA4KJXLYH | NDAR_INVFJP3171C | NDAR_INVM03FUB7G | NDAR_INVU697JENW | NDAR_INVZJUBWF3F |
| NDAR_INV4XEMVP0N | NDAR_INVA4MH2U9C | NDAR_INVFJTY1HTY | NDAR_INVM05MNA24 | NDAR_INVU6LKLMJU | NDAR_INVZJUUZAPJ |
| NDAR_INV4XT06HUA | NDAR_INVA4N4XMZ4 | NDAR_INVFK4HG4H6 | NDAR_INVM0706PEX | NDAR_INVU6TK1ULB | NDAR_INVZJYGP0WE |
| NDAR_INV4XWZ2LJE | NDAR_INVA4PJGHJV | NDAR_INVFL02R0H4 | NDAR_INVM0JXYXX1 | NDAR_INVU710KUDX | NDAR_INVZKDXUC63 |
| NDAR_INV4YD2ARWH | NDAR_INVA4PY09B5 | NDAR_INVFLCZ4FD7 | NDAR_INVM0X5DYN0 | NDAR_INVU7276YU8 | NDAR_INVZKJXBJMM |
| NDAR_INV4YKPPGKA | NDAR_INVA4ZHJBK2 | NDAR_INVFLEN32ED | NDAR_INVM1HAJGWE | NDAR_INVU745HDPT | NDAR_INVZL4VD4CA |
| NDAR_INV4Z38T1RY | NDAR_INVA52KHGAJ | NDAR_INVFLGC9XLH | NDAR_INVM1Z5GMD8 | NDAR_INVU7ARUX6H | NDAR_INVZL9DL58E |
| NDAR_INV4Z96NDW1 | NDAR_INVA5GXM81U | NDAR_INVFLP37NUM | NDAR_INVM22URD3V | NDAR_INVU7DNCYGM | NDAR_INVZM3MAF8B |
| NDAR_INV4Z9RE0CC | NDAR_INVA5YLKP02 | NDAR_INVFLVCCKDN | NDAR_INVM255ZJ8M | NDAR_INVU7VBC2CJ | NDAR_INVZM7EZFZF |
| NDAR_INV4ZAN961T | NDAR_INVA62ZL91V | NDAR_INVFM8FB6B7 | NDAR_INVM2EJE47J | NDAR_INVU8D0FE3D | NDAR_INVZMACPUER |
| NDAR_INV4ZMG12VE | NDAR_INVA66XU98A | NDAR_INVFMCMJ0X2 | NDAR_INVM2FL3HW3 | NDAR_INVU8D4R4YF | NDAR_INVZMMCVRWG |
| NDAR_INV4ZMTGFKA | NDAR_INVA6LB7NLM | NDAR_INVFP0ZD9PP | NDAR_INVM2JLZJJ1 | NDAR_INVU99HJDM4 | NDAR_INVZMVK0FMA |
| NDAR_INV4ZPG6HXX | NDAR_INVA776K15W | NDAR_INVFPC3YFJZ | NDAR_INVM2R7LWZ1 | NDAR_INVU9C36KFY | NDAR_INVZMZKERHD |
| NDAR_INV4ZTRVWH5 | NDAR_INVA7CLY4PL | NDAR_INVFPFM6B57 | NDAR_INVM3BPA3ZR | NDAR_INVU9C9ZMKM | NDAR_INVZN4F9J96 |
| NDAR_INV507WXWN7 | NDAR_INVA7PB1MWM | NDAR_INVFPLM1B6G | NDAR_INVM42YXRFM | NDAR_INVU9DAJLMN | NDAR_INVZN9E45NT |
| NDAR_INV50A1BC7T | NDAR_INVA850LWF0 | NDAR_INVFPXXMVAT | NDAR_INVM48HLU0M | NDAR_INVU9MU0V55 | NDAR_INVZN9E7J06 |
| NDAR_INV50C4P125 | NDAR_INVA8J2VT49 | NDAR_INVFR8VJPEX | NDAR_INVM4D87T1B | NDAR_INVU9VKB2N8 | NDAR_INVZNBXJ0R7 |
| NDAR_INV50H55Y7T | NDAR_INVA8JXUDR3 | NDAR_INVFR9N4HJY | NDAR_INVM4MC3HPC | NDAR_INVUAERPXD7 | NDAR_INVZNN6JK3K |
| NDAR_INV50K76F6L | NDAR_INVA940A4NE | NDAR_INVFRD95FDC | NDAR_INVM4ZC6HW9 | NDAR_INVUAYNYWT7 | NDAR_INVZNTJH2GT |
| NDAR_INV50M00BEW | NDAR_INVA9CA7RPB | NDAR_INVFRW3A0M2 | NDAR_INVM50LU9VD | NDAR_INVUB22FPTE | NDAR_INVZNW7Y6R1 |
| NDAR_INV50P8MAGW | NDAR_INVA9YBPFCH | NDAR_INVFT0UZ37W | NDAR_INVM5F9LGBU | NDAR_INVUB8UKMXG | NDAR_INVZNXT0TX2 |
| NDAR_INV50UAMBFC | NDAR_INVA9ZW33TZ | NDAR_INVFTCMGAD3 | NDAR_INVM5P36WUD | NDAR_INVUBLUW5AM | NDAR_INVZP36MY9W |
| NDAR_INV50UATGZM | NDAR_INVAADDZK5V | NDAR_INVFTEDMTCZ | NDAR_INVM5VGRR57 | NDAR_INVUC80Z0WM | NDAR_INVZPEYC15U |
| NDAR_INV51B6T1FL | NDAR_INVAB55U5W2 | NDAR_INVFTKHAA7R | NDAR_INVM6LGY18K | NDAR_INVUCF0VBM1 | NDAR_INVZR9NMJBR |
| NDAR_INV51EYYMEY | NDAR_INVABCY2GG6 | NDAR_INVFUMZH19F | NDAR_INVM6LR22KX | NDAR_INVUCGJ1U8F | NDAR_INVZRLLR2ZX |
| NDAR_INV51GZ9MPP | NDAR_INVABKRZFC9 | NDAR_INVFVBUVXGF | NDAR_INVM6VG3Z7Y | NDAR_INVUCUU472H | NDAR_INVZRR4D9LW |
| NDAR_INV51ZUA0EC | NDAR_INVABLFF21T | NDAR_INVFVFMMHD7 | NDAR_INVM6XB1GYE | NDAR_INVUCXEZB27 | NDAR_INVZT7CGM7G |
| NDAR_INV51ZUKMA3 | NDAR_INVACLWDDC2 | NDAR_INVFVK6MXVG | NDAR_INVM7175YDC | NDAR_INVUDBJ9V2N | NDAR_INVZTFAZ4PF |
| NDAR_INV52034BGR | NDAR_INVAD301F7N | NDAR_INVFVKBTZUT | NDAR_INVM7T4Y22R | NDAR_INVUDEPUVAK | NDAR_INVZTHGEAWM |
| NDAR_INV52AE3MBX | NDAR_INVADWJCAR7 | NDAR_INVFVN56Y3J | NDAR_INVM7YD6387 | NDAR_INVUDUKRV36 | NDAR_INVZTN5R70A |
| NDAR_INV52CVLNFF | NDAR_INVAE0K8UGX | NDAR_INVFVP5BP99 | NDAR_INVM86N4GZV | NDAR_INVUE7CU1V9 | NDAR_INVZUN3P64X |
| NDAR_INV52PY7ZPT | NDAR_INVAEAYWLPJ | NDAR_INVFVT2Z4U9 | NDAR_INVM8824V2L | NDAR_INVUEBPZ7X3 | NDAR_INVZUXFF006 |
| NDAR_INV52UNJAGX | NDAR_INVAEMLZKT4 | NDAR_INVFVXF1HTH | NDAR_INVM892T4X1 | NDAR_INVUELN86MB | NDAR_INVZV54BWRE |
| NDAR_INV533LN4K1 | NDAR_INVAER4MX1D | NDAR_INVFVY5PYH7 | NDAR_INVM8HMUPN5 | NDAR_INVUET7ENDU | NDAR_INVZVD13ZMG |
| NDAR_INV53BA6MNE | NDAR_INVAF36HM7X | NDAR_INVFWC6LDUD | NDAR_INVM8LL41UF | NDAR_INVUFF64VGJ | NDAR_INVZVW3HKWN |
| NDAR_INV53CVF7NB | NDAR_INVAF517NF3 | NDAR_INVFWFC9K4K | NDAR_INVM8PMJ7MH | NDAR_INVUGD7DXX7 | NDAR_INVZWE6CD6Y |
| NDAR_INV53EP1G5X | NDAR_INVAF722ZVJ | NDAR_INVFWU0HW96 | NDAR_INVM91VXF6F | NDAR_INVUGKLRP0A | NDAR_INVZWP490TG |
| NDAR_INV53L8E8LF | NDAR_INVAFCVVYJ2 | NDAR_INVFWYFBEK0 | NDAR_INVM96YWHXB | NDAR_INVUGKWA479 | NDAR_INVZWRJXNHE |
| NDAR_INV53LYW9M5 | NDAR_INVAFNHCVUJ | NDAR_INVFX004DUH | NDAR_INVM99BLZUE | NDAR_INVUGP7XGXD | NDAR_INVZWWDT1TG |
| NDAR_INV546G53XG | NDAR_INVAFZ44PA8 | NDAR_INVFX2LPCN8 | NDAR_INVM9KC36N1 | NDAR_INVUGP8FHFC | NDAR_INVZXKWKTC7 |
| NDAR_INV54DNDTDX | NDAR_INVAG1DBDKY | NDAR_INVFXBP5YUC | NDAR_INVM9VTUE2G | NDAR_INVUH0F2U1W | NDAR_INVZY3TE53A |
| NDAR_INV54HNXP3N | NDAR_INVAGCC9200 | NDAR_INVFXC7A0T2 | NDAR_INVMA0MXL96 | NDAR_INVUH77PVBN | NDAR_INVZYFB43H7 |
| NDAR_INV54WD6UV3 | NDAR_INVAGGKXW0P | NDAR_INVFXG8Z1V0 | NDAR_INVMA6DD9GU | NDAR_INVUHCPUV4G | NDAR_INVZYLV9BMB |
| NDAR_INV556U6DJ0 | NDAR_INVAH92KG8X | NDAR_INVFXLHXVKE | NDAR_INVMAABD7MX | NDAR_INVUHEK7K6F | NDAR_INVZZ05KY5J |
| NDAR_INV55BK5ZXT | NDAR_INVAHDU5HRW | NDAR_INVFY2JWCBT | NDAR_INVMABFTW07 | NDAR_INVUJBHEFTN | NDAR_INVZZ35KHXP |
| NDAR_INV55HCZHHV | NDAR_INVAHREHU85 | NDAR_INVFYK5K5G7 | NDAR_INVMAK80V91 | NDAR_INVUJGVYU5N | NDAR_INVZZL0VA2F |
| NDAR_INV55R9F7EX | NDAR_INVAHUKGDEE | NDAR_INVFYNEZGWE | NDAR_INVMAZCGHX3 | NDAR_INVUKDUJYJH | NDAR_INVZZNX6W2P |
| NDAR_INV569JK9GU | NDAR_INVAJ78XD39 | NDAR_INVFYUU1CWH | NDAR_INVMB7HFCAV | NDAR_INVUKKJ04AZ | NDAR_INVZZZP87KR |
| NDAR_INV56KML05T | NDAR_INVAJBZT1BK | NDAR_INVFZ16A6M3 | NDAR_INVMBD8R833 | NDAR_INVUKPZU1JW |  |
| NDAR_INV56L41G67 | NDAR_INVAJU25405 | NDAR_INVFZ1W3N0D | NDAR_INVMC12A6UB | NDAR_INVULE1VKVR |  |
| NDAR_INV56VJH1CR | NDAR_INVAK35AT6W | NDAR_INVFZ4F6Y38 | NDAR_INVMC1L92VA | NDAR_INVULEGZ5EL |  |
